# Validation of a Risk Score for Cancer-Associated Thrombosis Using Nationwide EHR Data

**DOI:** 10.1001/jamanetworkopen.2025.44428

**Published:** 2025-11-25

**Authors:** Ang Li, Omid Jafari, Barbara D. Lam, Jun Y. Jiang, Rock Bum Kim, Shengling Ma, Emily Zhou, Joyce W. Tiong, Elizabeth C. Chiang, Justine Ryu, Christopher I. Amos, Jennifer La, Nathanael R. Fillmore

**Affiliations:** 1 Section of Hematology-Oncology, Baylor College of Medicine, Houston, Texas; 2 Division of Hematology & Oncology, Fred Hutch Cancer Center, University of Washington, Seattle; 3 McGovern Medical School, University of Texas Health Science Center at Houston; 4 School of Medicine, Baylor College of Medicine, Houston, Texas; 5 Department of Medicine, Section of Hematology, Yale School of Medicine, New Haven, Connecticut; 6 Population Sciences and Cancer Control, University of New Mexico, Alburquerque; 7 Massachusetts Veterans Epidemiology Research and Information Center, VA Boston Healthcare System, Boston, Massachusetts; 8 VA Boston Healthcare System, Harvard Medical School, Boston, Massachusetts

## Abstract

**Importance:**

Venous thromboembolism (VTE) is associated with increased mortality and morbidity in patients with cancer. Existing risk prediction models are typically validated within individual sites, a fragmented approach that limits clinical adoption.

**Objective:**

To validate the electronic health record cancer-associated thrombosis (EHR-CAT) score compared with the benchmark Khorana score in a contemporary cohort of patients with cancer across the nation, before and after treatment, excluding those at high risk of bleeding.

**Design, Setting, and Participants:**

This prognostic study included patients in a nationwide longitudinal EHR database from January 2018 to December 2023 with follow-up continuing to April 2025. Patients with newly diagnosed, invasive, solid, or hematologic malignant neoplasms (defined using validated *International Statistical Classification of Diseases, Tenth Revision, Clinical Modification [ICD-10-CM]* algorithms) receiving systemic therapy (defined using the first antineoplastic medication) were included. Those with recent history of acute VTE diagnosis or anticoagulant prescription were excluded.

**Exposures:**

Demographics, risk model variables, and common anticoagulant trial exclusion criteria (as a proxy for identifying people at high risk of bleeding) were extracted on or before index therapy initiation date.

**Main Outcomes:**

Incident VTE and bleeding outcomes at 6 months were defined using validated *ICD-10-CM* algorithms.

**Results:**

A total of 732 594 patients (median [IQR] age, 65.0 [56.9-73.0] years; 425 124 female [58.0%]; 25 634 Asian [3.5%], 94 269 Black [12.9%], 48 266 Hispanic [6.6%], 583 047 White [76.9%]) with active cancer receiving systemic therapy between 2018 and 2023 from 184 health systems were identified. With a median (IQR) follow-up of 676 (340-1151) days, the incidence of 6-month VTE, bleeding, and mortality was 4.7% (34 499 patients), 3.7% (26 993 patients), and 8.4% (60 239 patients), respectively. Bleeding risk was higher in the 26.0% of patients (190 413) meeting anticoagulant trial exclusion criteria (7.2% vs 2.4%; hazard ratio, 2.5 [95% CI, 2.5-2.5]). The EHR-CAT score stratified patients into discriminative risk groups (C statistic, 0.70-0.71) both before and after exclusion for bleeding risk. When compared with the benchmark Khorana score (C statistic, 0.63), EHR-CAT reclassified 20% of patients into revised categories with improved prediction accuracy. Furthermore, EHR-CAT had consistent calibration in subgroups by age, sex, race, ethnicity, and individual health system sites.

**Conclusions:**

This prognostic study of the EHR-CAT risk score demonstrated the external validity and feasibility of using readily available structured EHR data to estimate VTE risk in patients with cancer.

## Key Points

**Question:**

Can routinely collected electronic health record (EHR) data from diverse health systems be used to model cancer-associated thrombosis (CAT) risk?

**Findings:**

In this prognostic study using a retrospective cohort of 732 594 patients with cancer receiving systemic therapy between 2018 and 2023 from 184 health systems, the EHR-CAT score significantly outperformed the benchmark Khorana score and had 20% improved accuracy. The model had consistent calibration by demographic subgroups, health system sites, and cohorts stratified by bleeding risk.

**Meaning:**

These results suggest that standardized structured EHR data from different health systems can support scalable validation and implementation of CAT risk assessment.

## Teaser

This prognostic study of US patients with cancer receiving systemic therapy validates an electronic health record–based risk prediction model for mortality and morbidity associated with venous thromboembolism.

## Introduction

Cancer-associated venous thromboembolism (VTE), particularly pulmonary embolism and lower extremity deep vein thrombosis (LE-DVT), is associated with increased risk of death and complications in patients with active cancer.^1,2^ Various society guidelines suggest low-dose thromboprophylaxis in patients with cancer who are deemed high risk for thrombosis by a validated risk assessment tool.^3,4^ The Khorana score, initially derived in 2008, has been the most cited benchmark model,^5^ but the proportion of VTE cases captured in the high-risk group varies between 23% and 55%.^6^ Newer models, including our own electronic health record cancer-associated thrombosis (EHR-CAT) risk score,^7^ have incorporated more clinical covariates to improve model accuracy and discrimination, albeit at the potential cost of increased complexity. Currently, most oncology clinicians do not perform VTE risk assessments or prescribe thromboprophylaxis to their high-risk patients.^8,9^

The implementation of VTE risk assessment and prevention in cancer has been hindered by several challenges. The variables required to calculate risk scores are not consistently available or standardized across electronic health record (EHR) systems, making wide-scale external validation and adoption difficult. Bleeding risk is also a critical consideration for patients with cancer, but there are no clear criteria for identifying high-risk patients. These challenges are especially pronounced in the US, where fragmented EHR systems limit the assessment of risk models as well as the ability to accurately track national patterns in cancer-related complications.

In this study, we used a contemporary, longitudinal EHR database that incorporated data from numerous health systems and defined a nationwide cohort of patients with active cancer receiving systemic therapy over a 6-year period. Using objective and readily available structured EHR data, we extracted baseline covariates for the VTE risk scores and identified patient characteristics aligned with anticoagulant trial exclusion criteria as a proxy for bleeding risk. We evaluated the feasibility and performance of the VTE risk scores before and after excluding patients at high risk for bleeding.

## Methods

### Data Source

We used retrospective data collected by Epic Cosmos, a dataset created in collaboration with a community of Epic EHR health systems representing more than 298 million patients from over 1711 hospitals and 39 900 clinics nationwide.^21^ Linked and deidentified data was made available in the Expertly Determined De-Identified (EDDI) data set. The current analysis was performed using the April 9, 2025, EDDI data refresh and included patients with active cancer receiving systemic therapy from January 2018 through December 2023 with a lookback window to January 1, 2017, and a follow-up truncation on April 1, 2025.

The study was deemed non–human participants research by the institutional review board at Baylor College of Medicine. The analytic plan and reporting followed the Transparent Reporting of a Multivariable Prediction Model for Individual Prognosis or Diagnosis (TRIPOD) reporting guideline checklist for prediction model validation.

### Participants

Full details are in eMethods and eTables 1 through 3 in Supplement 1. Briefly, health systems were required to be US based, validated for data completeness, and included a minimum frequency of face-to-face encounters in hematology and oncology departments to ensure the organization contributed complete, continuous, and cancer-relevant EHR data. From each eligible site, newly diagnosed cancers were defined using validated *International Classification of Diseases, Tenth Edition, Clinical Modification* (*ICD-10-CM*) coding algorithm.^7^ Cohort inclusion criteria included adults with incident cancer diagnosis with at least 1 new systemic therapy. Cohort exclusion criteria included acute VTE diagnosis or active anticoagulant prescription in the last 12 months before index date to ensure the cohort was not affected by those with recent outcome exposure or receiving anticoagulant prophylaxis. A subcohort was created for sensitivity analysis to exclude patients at risk for bleeding based on anticoagulation clinical trial exclusion criteria.^11,12^ These criteria included acute leukemia, primary or metastatic brain tumor, recent history of bleeding, platelet and/or alanine aminotransferase levels more than 5 times the upper limit, bilirubin more than 2 times the upper limit, glomerular filtration rate (GFR) below 30 mL/min/1.73 m^2^, weight less than 40 kg, anticoagulants, nonaspirin antiplatelet drugs, or drugs with strong CYP3A4 interactions.

### Outcomes

The primary outcome was incident overall VTE, which was defined as the first occurrence of acute pulmonary embolism, LE-DVT, or upper extremity (UE) DVT. Secondary outcomes included acute pulmonary embolism or LE-DVT, all-cause mortality, and bleeding. Patients were followed from the index date of systemic therapy initiation until an outcome event, the censor date (last face-to-face encounter before a 6-month encounter-free gap), death, or April 1, 2025, whichever came first. All outcomes were defined using validated *ICD-10-CM* codes with 95% or more precision. Full outcome definitions and validation metrics are in eMethods and eTable 4 in Supplement 1.

### Predictors

Baseline data encompassed demographic, clinical, and laboratory variables, including 26 cancer types and 4 systemic therapy types. Detailed information defining each variable is given in eMethods and eTable 5 in Supplement 1. Definitions and thresholds for EHR-CAT and Khorana score are described in eTable 6 in Supplement 1. Both risk models included body mass index (BMI; calculated as weight in kilograms divided by height in meters squared) and complete blood count (CBC). In addition to reclassified cancer types, the former risk model contained 6 additional variables (advanced stage, history of VTE, history of paralysis, recent hospitalization, targeted or endocrine monotherapy, Asian or Pacific Islander race). EHR-CAT was grouped into 6 categories (0 or less, 1, 2, 3, 4, 5 or more) and Khorana score was grouped into 4 categories (0, 1, 2, 3 or more) according to initial model development suggestions.^5,7^

### Statistical Analysis

To ensure accurate external validation, we assessed the model performance using the sum of integer scores without model updating or recalibration. Specifically, discrimination was evaluated using time-dependent receiver operating characteristic (ROC) curve (C statistic) and bootstrapped 95% CIs.^10^ Calibration was evaluated using cumulative incidence calibration plots and compared with the incidence at each risk score from the initial derivation cohort. Patients with missing BMI and CBC strongly correlated with early-stage breast and prostate cancer and adjuvant endocrine therapy. Adhering to the derivation study, we excluded these patients as they would not typically be considered for thromboprophylaxis. Metastatic *ICD-10-CM* codes were used to define advanced stage when documented stage was missing. There were no missing data in the remaining variables, and no imputation was performed. Key sensitivity analysis was performed after excluding patients at risk for bleeding based on anticoagulant clinical trial exclusion criteria.^11,12^ Additional subgroup analyses were done by age, sex, self-reported race, ethnicity, and individual site to ensure model fairness. All analyses were performed using R version 4.4.3 (R Project for Statistical Computing).

## Results

### Organizations and Participants

**Figure 1.**
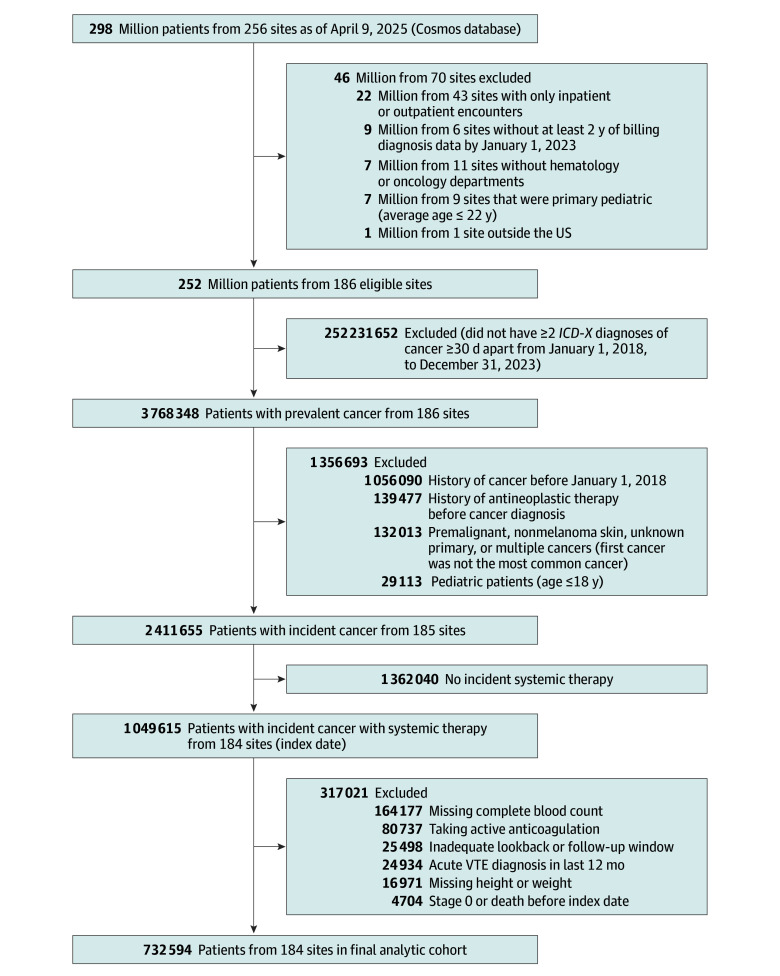
Cohort Selection From Cosmos *ICD-10-CM* indicates *International Classification of Diseases, Tenth Edition*, Clinical Modification; VTE, venous thromboembolism.

As of April 9, 2025, there were 298 million unique patients from 256 sites in Cosmos. After organizational exclusion to ensure high-quality, longitudinal, and cancer-relevant data, 252 million patients from 186 health systems in all 50 states remained eligible for cohort selection. Among these sites, 185 had 3 or more years of data, 164 had 5 or more years of data, and 150 had 7 or more years of data. From eligible health systems, we identified 2 411 655 patients with newly diagnosed cancer and 1 050 833 patients who started new systemic therapy after diagnosis from 184 sites between January 2018 and December 2023. After further cohort exclusion, 732 594 patients with active cancer receiving systemic therapy remained in the primary analytic cohort (Figure 1).

The median (IQR) age was 65.0 (56.9-73.0) years; 425 124 (58.0%) were female (Table 1). Self-reported race included 25 634 Asian or Pacific Islander (3.5%), 94 269 Black (12.9%), and 583 047 White (79.6%) patients; 48 266 (6.6%) had Hispanic ethnicity, and 144 926 (19.8%) lived in rural or micropolitan areas. The median (IQR) social vulnerability index (SVI) was 0.6 (0.3-0.8), and the median (IQR) National Cancer Institute Comorbidity Index was 0.3 (0-0.7). The most common cancers were breast (205 298 [28.0%]), lung (83 208 [11.4%]), colorectal (59 852 [8.2%]), and prostate (51 589 [7.0%]). Cancer stage was 123 806 patients (16.9%) with stage I to II disease, 58 126 (7.9%) with stage III disease, 206 334 (28.2%) with stage IV or metastatic disease, and 119 956 (16.4%) with unstageable disease (brain or leukemia). Treatment included 446 048 patients (60.9%) receiving cytotoxic chemotherapy, 156 644 (21.4%) receiving endocrine therapy, 81 987 (11.2%) receiving targeted therapy, and 47 915 (6.5%) immune checkpoint inhibitor therapy. The median (IQR) time between first cancer diagnosis encounter and therapy initiation was 35 (14-77) days.

**Table 1.** Baseline Patient Characteristics and Risk Score Assignments

Characteristic	Patients, No. (%) (N = 732 594)	EHR-CAT	Khorana	Bleed risk^a^
Age, median (IQR), y	65.0 (56.9-73.0)	0	0	0
Sex				
Female	425 124 (58.0)	0	0	0
Male	307 430 (42.0)	0	0	0
Self-reported race				
Asian or Pacific Islander	25 634 (3.5)	−1	0	0
Black	94 269 (12.9)	0	0	0
White	583 047 (79.6)	0	0	0
Other or unknown^b^	29 644 (4.0)	0	0	0
Ethnicity				
Non-Hispanic	663 433 (90.6)	0	0	0
Hispanic	48 266 (6.6)	0	0	0
Unknown	20 895 (2.9)	0	0	0
Rural-urban commuting area				
Metropolitan	585 625 (79.9)	0	0	0
Micropolitan	75 499 (10.3)	0	0	0
Rural	69 427 (9.5)	0	0	0
Unknown	2043 (0.3)	0	0	0
Social Vulnerability Index, median (IQR)	0.6 (0.3-0.8)	0	0	0
NCI Comorbidity Index, median (IQR)	0.3 (0-0.7)	0	0	0
Cancer type				
Breast	205 298 (28.0)	0	0	0
Prostate	51 589 (7.0)	0	0	0
Lung	83 208 (11.4)	+2	+1	0
Lower GI^c^	59 852 (8.2)	+1	0	0
Upper GI^d^	19 257 (2.6)	+3	+2	0
Pancreas	24 055 (3.3)	+3	+2	0
Bile and gallbladder	8040 (1.1)	+3	0	0
Liver	8465 (1.2)	0	0	0
Head and neck	29 782 (4.1)	0	0	0
Bladder	14 612 (2.0)	+2	+1	0
Kidney	11 625 (1.6)	+2	+1	0
Testis	4078 (0.6)	+2	+1	0
Uterus	16 928 (2.3)	+2	+1	0
Ovary	14 571 (2.0)	+2	+1	0
Cervix	8005 (1.1)	0	+1	0
Brain	16 110 (2.2)	+2	0	1
Melanoma	13 915 (1.9)	0	0	0
Sarcoma	5439 (0.7)	+2	0	0
Myeloma	24 344 (3.3)	+2	0	0
Lymphoma (aggressive)^e^	26 094 (3.6)	+2	+1	0
Lymphoma (indolent)^f^	22 481 (3.1)	0	+1	0
Leukemia (AML)	10 692 (1.5)	0	0	1
Leukemia (ALL)	3285 (0.4)	+2	0	1
Leukemia (CML, MDS)	16 728 (2.3)	0	0	0
Leukemia (CLL)	11 926 (1.6)	0	0	0
Other cancer	22 215 (3.0)	0	0	0
Cancer stage				
Stage I	79 368 (10.8)	0	0	0
Stage II	44 438 (6.1)	0	0	0
Stage III	58 126 (7.9)	+1	0	0
Stage IV	60 700 (8.3)	+1	0	0
Metastatic cancer *ICD-10-CM* code^g^	145 634 (19.9)	+1	0	0
Metastatic brain *ICD-10-CM* code^g^	20 974 (2.9)	0	0	1
Unstageable^h^	119 956 (16.4)	0	0	0
Unknown	224 372 (30.6)	0	0	0
Therapy type				
Cytotoxic chemotherapy	446 048 (60.9)	0	0	0
Immune checkpoint inhibitor	47 915 (6.5)	0	0	0
Targeted therapy	81 987 (11.2)	−1	0	0
Endocrine therapy	156 644 (21.4)	−1	0	0
BMI				
Continuous	27.5 (23.8 to 32.2)	0	0	0
≥35	113 333 (15.5)	+1	+1	0
Weight <40 kg	2412 (0.3)	0	0	1
Historical diagnoses				
Hospitalization last 3 mo	171 145 (23.4)	+1	0	0
Paralysis last year	9891 (1.4)	+1	0	0
Chronic VTE last year^i^	23 474 (3.2)	+1	0	0
Bleeding last year	96 907 (13.2)	0	0	1
Active or reported prescription				
Anticoagulant (stopped)^j^	3350 (0.5)	0	0	1
Antiplatelet (nonaspirin)	17 419 (2.4)	0	0	1
CYP3A4-interacting drug	12 667 (1.7)	0	0	1
White blood cell				
Median (IQR), cells/μL	7200 (5600-9500)	0	0	0
Count >11 000 cells/μL	117 748 (16.1)	+1	+1	0
Unknown	0	0	0	0
Hemoglobin				
Median (IQR), g/dL	12.6 (11.0-13.8)	0	0	0
Levels <10 g/dL	105 419 (14.4)	+1	+1	0
Unknown	0	0	0	0
Platelet				
Median (IQR), 10^3^/μL	249.0 (196.0-312.0)	0	0	0
Count ≥350 × 10^3^/μL	117 572 (16.0)	+1	+1	0
Count <50 × 10^3^/μL	14 349 (2.0)	0	0	1
Unknown	0	0	0	0
Alanine transaminase				
Median (IQR), units/L	20.0 (14.0-29.0)	0	0	0
Levels ≥260 units/L	2074 (0.3)	0	0	1
Unknown	50 843 (6.9)	0	0	0
Total bilirubin				
Median (IQR), mg/dL	0.5 (0.3-0.7)	0	0	0
Levels ≥2.4 mg/dL	7176 (1.1)	0	0	1
Unknown	68 744 (9.4)	0	0	0
Creatinine				
Median (IQR), mg/dL	0.8 (0.7-1.0)	0	0	0
eGFR levels <30 mL/min/1.73 m^2^	17 708 (2.5)	0	0	1
Unknown	32 056 (4.4)	0	0	0

Abbreviations: ALL, acute lymphoblastic leukemia; AML, acute myeloid leukemia; BMI, body mass index (calculated as weight in kilograms divided by height in meters squared); CLL, chronic lymphocytic leukemia; CML, chronic myeloid leukemia; eGFR, estimated glomerular filtration rate; GI, gastrointestinal; *ICD-10-CM*, *International Classification of Diseases, Tenth Edition, Clinical Modification*; MDS, myelodysplastic syndrome; NCI, National Cancer Institute; VTE, venous thromboembolism.

SI conversion factor: to convert alanine transaminase to microkatal/L, multiply by 0.0167; bilirubin to micromoles/L, multiply by 17.104; creatinine to micromoles/L, multiply by 88.4; hemoglobin to g/L, multiply by 10; white blood cells to × 10^9^ per liter, multiply by 0.001.

^a^ This column indicates the variables that were considered when excluding patients at risk for bleeding for sensitivity analysis based on anticoagulation clinical trial exclusion criteria. These included acute leukemia, primary or metastatic brain tumor, recent history of bleeding, platelet count less than 105 × 10^3^/μL, alanine transaminase levels more than 5 times the upper limit, total bilirubin more than 2 times the upper limit, eGFR less than 30 mL/min/1.73 m^2^, weight less than 40 kg, anticoagulants, nonaspirin antiplatelet drugs, or drugs with strong CYP3A4 interactions.

^b^ Other includes American Indian or Alaska Native, other race, and unknown (missing).

^c^ Lower GI includes colorectal and intestinal cancers.

^d^ Upper GI includes gastric and esophageal cancers.

^e^ Aggressive lymphoma includes diffuse large B-cell lymphoma, Burkitt lymphoma, lymphoblastic lymphoma, systemic T/natural killer-cell lymphoma.

^f^ Indolent lymphoma includes follicular lymphoma, mantle cell lymphoma, cutaneous T-cell lymphoma, Hodgkin lymphoma, and other lymphoma.

^g^ Metastatic cancer and metastatic brain are defined using *ICD-10-CM* codes (eTable 5 in Supplement 1).

^h^ Unstageable cancer includes all hematologic malignant neoplasms and brain cancer.

^i^ Only history/chronic VTE and those with acute event more than 1 year before index date is included (recent acute VTE is an exclusion criterion).

^j^ Only recent prescription of anticoagulation with a clear stop date before index date is included (recent anticoagulation initiation and active use is an exclusion criterion).

The allocation and distribution of individual VTE risk predictors for EHR-CAT and Khorana score are shown in Table 1. Notably, the 2 risk scores had distinct scoring metrics for cancer type. Shared risk predictors included BMI of 35 or higher (113 333 patients [15.5%]), white blood cell count above 11 000/μL (117 748 patients [16.1%]; to convert to cells × 10^9^ per liter, multiply by 0.001), hemoglobin levels below 10 g/dL (105 419 patients [14.4%]; to convert to grams per liter, multiply by 10), and platelet count of 350 × 10^3^/μL or higher (117 572 patients [16.0%]; to convert to cells × 10^9^, multiply by 1). Additional risk predictors for EHR-CAT included advanced stage (264 460 patients [36.1%]), targeted or endocrine therapy (156 644 patients [32.6%]), recent hospitalization (171 145 patients [23.4%]), recent paralysis or immobilization (9891 patients [1.4%]), remote VTE history (23 474 patients [3.2%]), and Asian or Pacific Islander race (25 634 patients [3.5%]). Furthermore, 190 413 patients (26.0%) would have met at least 1 exclusion criterion from anticoagulant clinical trials.

### Incidence of VTE and Bleeding Outcomes

With a median (IQR) continuous follow-up of 676 (340-1151) days, the 6-month incidences of overall VTE, pulmonary embolism, and DVT were 4.7% (34 499 patients) and 3.9% (28 341 patients), respectively. Nonexclusively, this represented 18 250 pulmonary embolism (2.5%), 15 658 LE-DVT (2.1%), and 7206 UE-DVT (1.0%) cases within 6 months. The incidence of VTE remained stable over 6 years (4.5% to 5.0%). With a median (IQR) follow-up of 1148 (688-1148) days for vital status, the 6-month overall mortality was 8.4% (60 239 patients).

The cumulative incidence of 6-month overall VTE and pulmonary embolism or LE-DVT by cancer type is shown in eFigure 1 in Supplement 1. Pancreatic (10.3% [2472 of 24 055 patients]), biliary or gallbladder (10.2% [822 of 8040 patients]), and acute lymphocytic leukemia (9.0% [298 of 3285 patients]) cancers had the highest 6-month incidence of VTE, while breast (2.0% [4072 of 205 298 patients]), chronic lymphocytic leukemia (1.8% [215 of 11 926 patients]), and prostate (1.7% [857 of 51 589 patients]) cancers had the lowest. Except for acute leukemias with higher incidence of catheter related UE-DVT, most cancers had correlated incidence of pulmonary embolism or LE-DVT and overall VTE.

The 6-month incidence of bleeding was 3.7% (26 993 patients). Clinical trial exclusion criteria were variably associated with bleeding risk, ranging from high (total bilirubin above 2.4 mg/dL [to convert to micromoles per liter, multiply by 17.104]: hazard ratio [HR], 2.78 [95% CI, 2.62-2.95]; acute leukemia: HR, 2.33 [95% CI, 2.22-2.44]; metastatic brain cancer: HR, 2.17 [95% CI, 2.09-2.26]; bleeding history: HR, 2.24 [95% CI, 2.20-2.29]; GFR <30 mL/min/1.73 m^2^: HR, 2.19 [95% CI, 2.11-2.29]) to low (alanine aminotransferase >260 units/L: HR, 1.32 [95% CI, 1.17-1.49]; strong CYP3A4 inducer/inhibitor: HR, 1.27 [95% CI, 1.21-1.34]; weight <40 kg: HR, 1.43 [95% CI, 1.25-1.62]; prior anticoagulant: HR, 1.42 [95% CI, 1.28-1.56]) (eTable 7 in Supplement 1). The incidence of 6-month bleeding was 2.4% in those with none of the exclusion criteria (541 181 patients) and 7.2% in those with any of them (190 413 patients) (HR, 2.55 [95% CI, 2.51-2.59]).

### Model Performance

**Figure 2.**
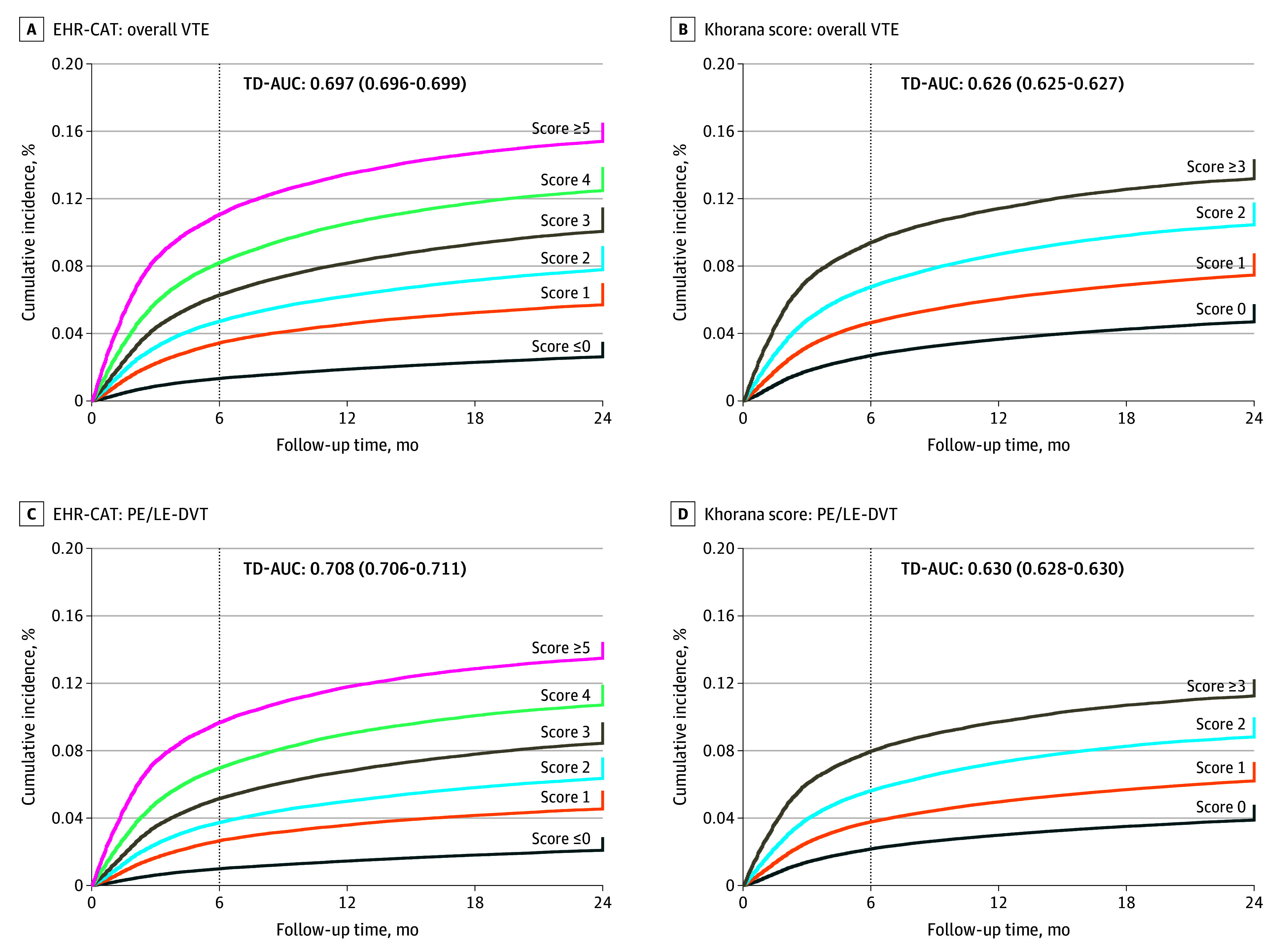
Performance of EHR-CAT vs Khorana Score EHR-CAT indicates electronic health record cancer-associated thrombosis; LE-DVT, lower extremity deep vein thrombosis; PE, pulmonary embolism; TD-AUC, time-dependent area of the receiver operating characteristic curve; VTE, venous thromboembolism.

We assessed the VTE risk scores’ performance in all eligible patients and among those deemed low bleeding risk from clinical trial exclusion criteria. In the primary analysis, the 6-month cumulative incidence of VTE when stratified by EHR-CAT was 1.3% for scores of 0 or less (3062 of 229 808 patients), 3.4% for a score of 1 (4059 of 117 796 patients), 4.7% for scores of 2 (5499 of 116 288 patients), 6.3% for scores of 3 (7096 of 112 900 patients), 8.2% for scores of 4 (7102 of 86 456 patients), and 11.1% for scores of 5 or more (7681 of 69 346 patients) (Table 2, Figure 2). The corresponding HRs (with 0 or less scores as reference) were 2.12 (95% CI, 2.05-2.18), 2.99 (95% CI, 2.92-3.11), 4.01 (95% CI, 3.90-4.12), 5.28 (95% CI, 5.14-5.44), and 7.31 (95% CI, 7.10-7.52). In contrast, the 6-month cumulative incidence of VTE when stratified by the Khorana score was 2.7% for scores of 0 (7447 of 275 609 patients), 4.7% for 1 (12 019 of 258 259 patients), 6.8% for 2 (9423 of 139 135 patients), and 9.4% for scores of 3 or more (5610 of 59 591 patients), respectively. The corresponding HRs (with 0 scores as reference) were 1.65 (95% CI, 1.61-1.68), 2.45 (95% CI, 2.40-2.51), and 3.37 (95% CI, 3.29-3.46). The pulmonary embolism and LE-DVT outcome followed a similar pattern in both models.

**Table 2.** Performance of EHR-CAT vs Khorana Score for VTE at 6 Months

Score	Cancer patients, No. (%) (N = 732 594)	Classification	VTE at 6 mo			PE or LE-DVT at 6 mo		
			No. (row %) (n = 34 499)	HR (95% CI)	TD-ROC	No. (row %) (n = 28 341)	HR (95% CI)	TD-ROC
EHR-CAT score								
≤0	229 808 (31.4)	Low risk	3062 (1.3)	1 [Reference]	0.70 (0.70-0.70)	2285 (1.0)	1 [Reference]	0.71 (0.71-0.71)
1	117 796 (16.1)		4059 (3.4)	2.12 (2.05-2.18)		3132 (2.7)	2.08 (2.01-2.15)	
2	116 288 (15.9)		5499 (4.7)	2.99 (2.92-3.11)		4350 (3.7)	3.01 (2.92-3.11)	
3	112 900 (15.4)	High risk	7096 (6.3)	4.01 (3.90-4.12)		5829 (5.2)	4.12 (3.99-4.25)	
4	86 456 (11.8)		7102 (8.2)	5.28 (5.14-5.44)		6033 (8.5)	5.53 (5.36-5.70)	
≥5	69 346 (9.5)		7681 (11.1)	7.31 (7.10-7.52)		6712 (9.7)	7.83 (7.59-8.08)	
Khorana score								
0	275 609 (37.6)	Low risk	7447 (2.7)	1 [Reference]	0.63 (0.63-0.63)	5989 (2.2)	1 [Reference]	0.63 (0.63-0.63)
1	258 259 (35.3)		12 019 (4.7)	1.65 (1.61-1.68)		9769 (3.8)	1.65 (1.61-1.69)	
2	139 135 (19.0)	High risk	9423 (6.8)	2.45 (2.40-2.51)		7829 (5.6)	2.49 (2.43-2.55)	
3+	59 591 (8.1)		5610 (9.4)	3.37 (3.29-3.46)		4754 (8.0)	3.44 (3.35-3.54)	

Abbreviations: HR, hazard ratio; LE-DVT, lower extremity deep vein thrombosis; TD-ROC, time-dependent receiver operating characteristic; VTE, venous thromboembolism.

For model discrimination, the time-dependent ROC (C statistic) for EHR-CAT was 0.697 (95% CI, 0.696-0.699) for overall VTE and 0.708 (95% CI, 0.706-0.711) for pulmonary embolism or LE-DVT (Figure 2). In contrast, the C statistic for the Khorana score was 0.626 (95% CI, 0.625-0.627) for overall VTE and 0.630 (95% CI, 0.628-0.630) for pulmonary embolism or LE-DVT. Similar to the derivation study, the EHR-CAT model outperformed the Khorana score by 0.07.^7^ For model calibration, we compared the observed with the predicted cumulative incidence of VTE at each score for EHR-CAT (eFigure 2 in Supplement 1). The model was well calibrated for pulmonary embolism or LE-DVT and modestly calibrated for overall VTE. We could not calibrate the Khorana score due to the lack of 6-month estimates from the initial study. When comparing the previously proposed clinical risk groups, EHR-CAT reclassified 20% of patients from the Khorana score groups into revised categories that showed improved concordance with the observed VTE risk (Table 3). The reclassification increased the total proportion of potentially preventable VTEs in the high-risk group from 43.6% to 63.4%.

**Table 3.** Comparison of EHR-CAT vs Khorana Score Risk Groups for VTE at 6 Months

Category	Khorana score	EHR-CAT	Cancer patients, No. (%) (N = 732 594)	No. (row %)	
				Overall VTE at 6 mo (n = 34 499)	PE or LE-DVT at 6 mo (28 341)
Concordant (80%)	Low risk	Low risk	423 899 (57.9)	11 355 (2.7)	8829 (2.1)
	High risk	High risk	158 733 (21.7)	13 768 (8.7)	11 645 (7.3)
Reclassified (20%)	Low risk	High risk	109 969 (15.0)	8111 (7.4)	6929 (6.3)
	High risk	Low risk	39 993 (5.5)	1265 (3.2)	938 (2.3)

Abbreviations: EHR-CAT, electronic health record cancer-associated thrombosis; LE-DVT, lower extremity deep vein thrombosis; PE, pulmonary embolism; VTE, venous thromboembolism.

### Sensitivity Analyses

Approximately 26% of the cohort met anticoagulant trial exclusion and had higher bleeding risk. In sensitivity analysis excluding these individuals (eTable 8 in Supplement 1), the 6-month incidence of VTE decreased from 4.7% to 4.2% and that of bleeding decreased from 3.7% to 2.4%; however, both risk scores retained similar covariate distribution and performance (C statistic: EHR-CAT, 0.71-0.72; Khorana score, 0.63-0.64).

We performed additional sensitivity analyses to ensure model fairness, generalizability, and interoperability. First, we performed subgroup analysis by age, sex, self-reported race, and ethnicity (eTable 9 in Supplement 1). EHR-CAT had similar discrimination in all subgroups as the primary analysis, except the male subgroup that had a slightly lower C statistic of 0.67 driven by the loss of breast cancer (28% of the cohort). Second, we randomly selected 10 health systems among the 135 sites that contributed over 1000 patients to demonstrate model performance on a site level (eTable 10 in Supplement 1). Each site had a uniquely different patient and cancer composition, and the 6-month VTE incidence varied from 3.5% and 6.2% (average, 4.8%), and the C statistic varied from 0.64 to 0.76 (average, 0.70). Finally, we attempted to simplify the model predictors to address the 2 variables with the most missingness. An exploratory multivariable analysis using individual variables is shown in eTable 11 in Supplement 1. When cancer stage was entirely substituted by metastatic *ICD-10-CM* codes, the C statistic was unchanged (0.70 for VTE). When all CBC values were removed from the model, the C statistic was slightly worse (0.69 for VTE).

## Discussion

Using an EHR database, we externally validated the performance and generalizability of VTE risk scores in a contemporary cohort of 732 594 patients with newly diagnosed cancer receiving systemic therapy from 2018 to 2023 across 184 health systems in the US. At 6 months after systemic therapy initiation, there were 4.7% overall VTE, 3.9% pulmonary embolism or LE-DVT, and 3.7% bleeding among patients with cancer. Using objective and extractable EHR data, the EHR-CAT risk score demonstrated a robust performance across different sites and accurately classified patients into groups with 6-month incidences of overall VTE ranging from 1.3% to 11.1% (C statistic of 0.70). Like the initial derivation study, the updated risk score outperformed the benchmark Khorana score by 0.07 (C statistic of 0.63) in the current analysis and reclassified 20% of patients into more appropriate risk groups. Finally, the EHR-CAT risk score performed well even after excluding 26% of patients at risk for bleeding using common anticoagulant exclusion criteria (C statistic of 0.70).

In contrast to previous validation studies,^13,14,15,16^ our current analytic cohort was large (732 594 patients), representative (all 50 states, with similar racial and ethnic demographics as the US census),^17^ contemporary (outcome ascertainment until April 2025), and with a median follow-up of 676 days. To ensure accuracy and external validity, we performed various data quality checks. The VTE and bleeding incidence remained similar across all years, indicating the absence of data shift and drift. The magnitude and pattern of VTE incidence across different cancer type, stage, and treatment was consistent with our previous study in male US veterans.^18^ The bleeding incidence at 2.4% after trial exclusion was also consistent with the 3.0% overall bleeding in the placebo arm of the CASSINI trial.^12^ Finally, the factors associated with VTE and bleeding were consistent with prior clinical knowledge.

While we defined baseline predictors and model fitting in the same way as the initial derivation studies, there were several notable differences in the sampling strategy that significantly improved its generalizability. First, we updated the study years from 2011-2020 to 2018-2024 and increased the number of sites from 1 to 184. Second, instead of relying on manually extracted cancer registry data to define cancer type, stage, and diagnosis date, we only used readily available structured data from the EHR. Third, we expanded systemic therapy initiation date to any time instead of only the first year after cancer diagnosis to ensure model generalizability. Fourth, we demonstrated that the model performed equally well in all patients as well as those deemed lower risk for bleeding using anticoagulant clinical trial exclusion criteria. This is important as the fear of bleeding and the challenge to identify patients who would qualify for clinical trials is often one of the key barriers to implementation.^8,9^ While not all trial exclusion criteria affected the bleeding risk in a similar magnitude, this was a starting point to build physician and patient trust for implementing the EHR-based VTE risk score. Finally, we showed that the risk score could have variations in performance when applied to single health systems (C statistic, 0.64 to 0.76), as it depended on patient composition. This likely explains the conflicting data on EHR-CAT’s absolute C statistic in previous studies.^13,14,15,16^

### Limitations

Our study has limitations due to the use of a deidentified, aggregated, and multi-institutional EHR database. First, while longitudinal data were joined in all the health systems that participate in Cosmos, interval missingness of follow-up data from non-participating institutions remained a source of error. To mitigate this, we censored patients who had a more than a 6-month gap from the previous face-to-face encounter, even if they had subsequent data. Second, missing data in baseline predictors could detract from the usability of any risk scores. Among the 11 predictors in EHR-CAT, the only 2 with significant missingness were cancer stage and CBC values. Fortunately, metastatic *ICD-10-CM* codes were a reasonable replacement for documented advanced cancer stage. For patients with missing CBC values, 70% had early-stage breast or prostate cancers and were receiving adjuvant endocrine therapy. We excluded this very low-risk group in the primary analysis; however, if we included these patients and imputed missing as low-risk for CBC values, the C statistic for EHR-CAT would have improved to 0.72. Third, given the lack of unstructured notes, we were not able to use an natural language processing algorithm to confirm VTE or bleeding cases. Therefore, we relied on previously validated *ICD*-based algorithms with relatively high positive predictive value (95%). The true incidence of outcome events could be underestimated, though the overall incidence was similar to previously published epidemiology studies.^18,19,20^ Finally, we acknowledge the complexity and accuracy trade-off of any risk scores. To reduce cost and facilitate implementation, we are actively working on translating our query into standardized database query that can be applied and embedded to EHR at each individual site.

## Conclusions

In summary, the EHR-CAT risk score performed well in discrimination and calibration of VTE outcomes in a contemporary cohort of patients with active cancer receiving systemic therapy, even after applying clinical trial exclusions to lower their bleeding risk. We further demonstrated the feasibility of using standardized and readily available structured data elements from EHR to calculate the risk score. With appropriate implementation, the EHR-CAT model has the potential to support thromboprophylaxis strategies across different health systems.

## Notes

Supplement 1. eMethods. eTable 1. Organization Filters eTable 2. Cohort Filters eTable 3. Systemic Therapy Classifications eTable 4. Outcome Filters eTable 5. Baseline Variables eTable 6. Detailed Predictor Definitions for EHR-CAT and Khorana Score eTable 7. Risk of Bleeding Based on Clinical Trial Exclusion Criteria eTable 8. Performance of EHR-CAT vs Khorana Score for VTE at 6 Months After Exclusion for Bleeding Risk eTable 9. Performance of EHR-CAT in Prespecified Subgroups eTable 10. Performance of EHR-CAT in 10 Randomly Selected Health Systems eTable 11. Exploratory Multivariable Cox Regression for EHR-CAT and Khorana Score Individual Predictors eFigure 1. Incidence of VTE at 6 Months by Cancer Type eFigure 2. Calibration Plots of EHR-CAT vs Original Derivation Model

Supplement 2. Data Sharing Statement
