# Supplementary material for: Validation of a Risk Score for Cancer-Associated Thrombosis Using Nationwide EHR Data
Source: JAMA Netw Open. 2025 Nov 25;8(11):e2544428. doi: 10.1001/jamanetworkopen.2025.44428 (PMC12648341; doi:10.1001/jamanetworkopen.2025.44428)
Supplement: Supplement 1. — eMethods. eTable 1. Organization Filters eTable 2. Cohort Filters eTable 3. Systemic Therapy Classifications eTable 4. Outcome Filters eTable 5. Baseline Variables eTable 6. Detailed Predictor Definitions for EHR-CAT and Khorana Score eTable 7. Risk of Bleeding Based on Clinical Trial Exclusion Criteria eTable 8. Performance of EHR-CAT vs Khorana Score for VTE at 6 Months After Exclusion for Bleeding Risk eTable 9. Performance of EHR-CAT in Prespecified Subgroups eTable 10. Performance of EHR-CAT in 10 Randomly Selected Health Systems eTable 11. Exploratory Multivariable Cox Regression for EHR-CAT and Khorana Score Individual Predictors eFigure 1. Incidence of VTE at 6 Months by Cancer Type eFigure 2. Calibration Plots of EHR-CAT vs Original Derivation Model [file jamanetwopen-e2544428-s001.pdf]

Supplemental Online Content

Li A, Jafari O, Lam BD, et al. Validation of a risk score for cancer-associated thrombosis using nationwide EHR data. *JAMA Netw Open.* 2025;8(11):e2544428. doi:10.1001/jamanetworkopen.2025.44428

eMethods.

eTable 1. Organization Filters

eTable 2. Cohort Filters

eTable 3. Systemic Therapy Classifications

eTable 4. Outcome Filters

eTable 5. Baseline Variables

eTable 6. Detailed Predictor Definitions for EHR-CAT and Khorana Score

eTable 7. Risk of Bleeding Based on Clinical Trial Exclusion Criteria

eTable 8. Performance of EHR-CAT vs Khorana Score for VTE at 6 Months After Exclusion for Bleeding Risk

eTable 9. Performance of EHR-CAT in Prespecified Subgroups

eTable 10. Performance of EHR-CAT in 10 Randomly Selected Health Systems

eTable 11. Exploratory Multivariable Cox Regression for EHR-CAT and Khorana Score Individual Predictors

eFigure 1. Incidence of VTE at 6 Months by Cancer Type

eFigure 2. Calibration Plots of EHR-CAT vs Original Derivation Model

This supplemental material has been provided by the authors to give readers additional information about their work.

© 2025 Li A et al. *JAMA Network Open*.

eMethods

Data Source

We used retrospective data collected by Epic Cosmos, a dataset created in collaboration with a community of Epic EHR health systems representing >298 million patients from over 1,711 hospitals and 39,900 clinics nationwide. The current counts for patients, hospitals, and clinics are available on cosmos.epic.com. Several high-impact clinical studies have been performed using the Cosmos database.[1–4] Longitudinal patient-level EHR data from multiple participating health systems were linked and deduplicated through the Care Everywhere network. Limited Data Sets were extracted every two weeks, curated, mapped to standard codes, and deidentified to remove unstructured texts and shift dates to create the Expertly Determined De-Identified (EDDI) Data Set. The current analysis was performed using the 4/9/2025 EDDI data refresh and included patients with active cancer receiving systemic therapy from 1/1/2018 to 12/31/2023 with a lookback window to 1/1/2017 and a follow-up truncation on 4/1/2025.

Participants

We created stringent organizational and cohort inclusion and exclusion criteria. Briefly, a health system was considered eligible if it was US-based; validated by Epic for data completeness; contributed billing diagnosis codes before 1/1/2023; had >1,000 face-to-face (F2F) inpatient, emergency department, and outpatient annual encounters; had >10,000 F2F annual encounters at hematology or oncology departments; and had an average patient age of >22 years (Supplemental Table 1). We imposed these filters to ensure the participating organization contributed complete, continuous, and cancer-relevant EHR data.

To define active cancer, we required each patient to have 2+ invasive cancer *International Classification of Diseases, Tenth Edition, Clinical Modification* (ICD-10-CM) codes as billing final or encounter diagnoses >30 days apart in selective F2F encounter and department types from 1/1/2018 to 12/31/2023 (Supplemental Table 2). Inpatient admissions and selective non-primary care outpatient departments were chosen to minimize historical cancer diagnosis. Furthermore, patients with non-melanomatous skin cancers, multiple cancer diagnoses where the initial cancer diagnosis was different from the most common one, premalignant conditions (e.g., polycythemia vera, essential thrombocythemia), unspecified or secondary metastatic ICD-10-CM codes without primary site, or previous cancer diagnosis codes or systemic therapy receipt within one year before first cancer diagnosis (including in the year 2017) were excluded.

To define systemic therapy, we extracted newly prescribed or administered antineoplastics medications after cancer diagnosis date using the therapeutic classification from the First Databank drug database (Supplemental Table 3). The medications were further classified as cytotoxic chemotherapy, immune checkpoint inhibitor, targeted therapy, or endocrine therapy. Therapeutic regimens were defined using all medications given within the first 4 weeks after the initial therapy. The index date was defined as the date of initial systemic therapy receipt.

To define the final analytic cohort, we applied additional lookback and follow-up exclusions from the index date of systemic therapy. Patients were further excluded if they did not have 1+ F2F encounters in the 12 months before the index date (for comorbidity) or 2+ F2F encounters after the index date (for outcome). Furthermore, patients were excluded for age <18 or >100 years, death date before index date, maximum cancer stage of

© 2025 Li A et al. *JAMA Network Open*.

0 (premalignant), active anticoagulant prescription started in the last 12 months, acute VTE diagnosis in the last 12 months, or missing body mass index (BMI) or complete blood count (CBC) in the last 3 months.

A sub-cohort was created for sensitivity analysis to exclude patients at risk for bleeding based on anticoagulation clinical trial exclusion criteria. These included acute leukemia, primary or metastatic brain tumor, recent history of bleeding, platelet $<150\times10^9$/L, alanine aminotransferase (ALT) >5x upper limit, bilirubin >2x upper limit, glomerular filtration rate (GFR) <30 mL/min/1.73 m^2, weight <40 kg, anticoagulants, non-aspirin antiplatelet drugs, or drugs with strong CYP3A4 interactions.

Outcomes
The primary outcome was incident overall VTE, which was defined as the first occurrence of acute PE, LE-DVT, or upper extremity (UE)-DVT (Supplemental Table 4). Secondary outcomes included acute PE/LE-DVT, all-cause mortality, and hospitalized bleeding. The death date and last known follow-up date were extracted directly from Cosmos. Patients were followed from the index date of systemic therapy initiation until an outcome event, the censor date (last F2F encounter before a 6-month encounter-free gap), death (if occurring no later than 3 months after the censor date), or 4/1/2025, whichever came first.

VTE was defined using our previously validated ICD-10-CM phenotyping algorithm from billing final diagnoses and encounter diagnoses using any inpatient F2F encounter or 2+ outpatient F2F encounters >30 to <365 days apart. Superficial venous thromboses, splanchnic vein thromboses,[5] septic emboli, and other atypical site thromboses were excluded as outcome events. This VTE algorithm was previously shown to have a positive predictive value [PPV] of 95%.[6]

Bleeding was defined using ICD-10-CM codes using billing final diagnoses from inpatient encounters only. To improve coding accuracy, we performed a systematic review of peer-reviewed manuscripts on ICD-10-CM bleeding codes and identified 545 non-duplicated codes from 6 relevant studies.[7–12] We then performed a two-physician independent review to adjudicate and remove ambiguous codes. A final list of 279 codes was included to define bleeding (Supplemental Table 4). Based on a random review of 100 patients at a single site, the algorithm had a PPV of 100% for clinically relevant bleeding (combined major and clinically relevant nonmajor bleeding)[13,14] and 72% for major bleeding alone.

Predictors
We defined baseline patient- and cancer-specific data, along with predictors for EHR-CAT and Khorana score models using variable lookback windows (3-12 months) (Supplemental Table 5). Baseline data extracted included age; sex; race; ethnicity; marital status; state; Rural-Urban Commuting Area code; Social Vulnerability Index (SVI); 26 consolidated cancer types; cancer stage; 4 systemic therapy types; 16 comorbidities (including paralysis) from the National Cancer Institute Comorbidity Index (NCI-CI);[15] height; weight; BMI; recent/current hospitalization of >3 days; CBC including white blood cell, hemoglobin, platelet; ALT; total bilirubin, creatinine, and GFR based on the Cockcroft-Gault formula; history of VTE (historic/chronic VTE any time or acute VTE >1 year before index date); history of bleeding; and use of anticoagulant, antiplatelet, and strong CYP3A4-inducing or -inhibiting medications. All laboratory features were defined using Logical Observation Identifiers, Names, and Codes (LOINC). Due to significant missingness in reported cancer stage (~50%), we further defined metastatic disease using ICD-10-CM codes. Advanced stage was subsequently determined as stages III-IV or having a metastatic ICD code.

© 2025 Li A et al. *JAMA Network Open*.

Individual binary predictor definitions and thresholds for the EHR-CAT and Khorana score models are described in Supplemental Table 6 (and https://li-lab-bcm.shinyapps.io/EHR-CAT/). Both risk models included BMI and CBC. In addition to reclassified cancer types, the former risk model contained 6 additional variables (advanced stage, history of VTE, history of paralysis, recent hospitalization, targeted or endocrine monotherapy, Asian or Pacific Islander race). EHR-CAT was grouped into 6 categories (0-, 1, 2, 3, 4, 5+) and Khorana score was grouped into 4 categories (0, 1, 2, 3+) according to initial model development suggestions.

Statistical Analysis

Cumulative incidence was estimated to account for the competing risk of death. To ensure accurate external validation, we assessed the model performance using the summation of rounded integer scores from each risk model without model updating or recalibration. Discrimination was evaluated using time-dependent receiver operating characteristic (ROC) curve (c statistic) and bootstrapped 95% confidence intervals.[16] Calibration was evaluated using cumulative incidence calibration plots and compared to the incidence at each risk score from the initial derivation cohort. Patients with missing BMI and CBC were mostly receiving endocrine therapy for early-stage breast or prostate cancers. We excluded these patients since they represented a group at an extremely low risk for VTE. Stage was missing in 51% with stageable cancers; therefore, we used metastatic ICD codes to supplement advanced stage. There were no missing data in the remaining variables, and no imputation was performed.

Sensitivity analyses were performed after excluding patients at risk for bleeding based on anticoagulation clinical trial exclusion criteria.[17,18] These included acute leukemia, primary or metastatic brain tumor, recent history of bleeding, platelet <$150x10^9$/L, ALT >260 units/L, bilirubin >2.4 mg/dL, GFR <30 mL/min/1.73 m^2, weight <40 kg, anticoagulants, non-aspirin antiplatelet drugs, or drugs with strong CYP3A4 interactions. Additional sensitivity analyses were performed by age, sex, race, and ethnicity subgroups to ensure model fairness. We also randomly selected 10 health systems to demonstrate model generalizability on the site level. All analyses were performed using R version 4.4.3 (R Foundation for Statistical Computing, Vienna, Austria). The study was approved by the Institutional Review Board at Baylor College of Medicine.

References

1. Moin EE, Seewald NJ, Halpern SD. Use of Life Support and Outcomes Among Patients Admitted to Intensive Care Units. *JAMA*. 2025;333(20):1793-1803. doi:10.1001/jama.2025.2163
2. Deputy NP, Deckert J, Chard AN, et al. Vaccine Effectiveness of JYNNEOS against Mpox Disease in the United States. *N Engl J Med*. 2023;388(26):2434-2443. doi:10.1056/NEJMoa2215201
3. Li P, Li Z, Staton E, et al. GLP-1 Receptor Agonist and SGLT2 Inhibitor Prescribing in People With Type 1 Diabetes. *JAMA*. 2024;332(19):1667-1669. doi:10.1001/jama.2024.18581
4. Kim C, Ross JS, Jastreboff AM, et al. Uptake of and Disparities in Semaglutide and Tirzepatide Prescribing for Obesity in the US. *JAMA*. Published online April 29, 2025. doi:10.1001/jama.2025.4735
5. Shang H, Jiang JY, Guffey D, et al. Natural history of cancer-associated splanchnic vein thrombosis. *Journal of Thrombosis and Haemostasis*. 2024;(October 2023):1-12. doi:10.1016/j.jtha.2024.01.019
6. Li A, Wilson L, Jr C, et al. Developing and optimizing a computable phenotype for incident venous thromboembolism in a longitudinal cohort of patients with cancer. *Res Pract Thromb Haemost*. 2022;6(4):e12733. doi:10.1002/rth2.12733

© 2025 Li A et al. *JAMA Network Open*.

7. Shehab N, Ziemba R, Campbell KN, et al. Assessment of ICD-10-CM code assignment validity for case finding of outpatient anticoagulant-related bleeding among Medicare beneficiaries. *Pharmacoepidemiol Drug Saf*. 2019;28(7):951-964. doi:10.1002/pds.4783
8. McDonald L, Sammon CJ, Samnaliev M, Ramagopalan S. Under-recording of hospital bleeding events in UK primary care: a linked Clinical Practice Research Datalink and Hospital Episode Statistics study. *Clin Epidemiol*. 2018;10:1155-1168. doi:10.2147/CLEP.S170304
9. Oger E, Botrel MA, Juchault C, Bouget J. Sensitivity and specificity of an algorithm based on medico-administrative data to identify hospitalized patients with major bleeding presenting to an emergency department. *BMC Med Res Methodol*. 2019;19(1):194. doi:10.1186/s12874-019-0841-6
10. Thaarup M, Nielsen PB, Olesen AE, et al. Positive Predictive Value of Non-Traumatic Bleeding Diagnoses in the Danish National Patient Register. *Clin Epidemiol*. 2023;15(April):493-502. doi:10.2147/CLEP.S400834
11. Joos C, Lawrence K, Jones AE, Johnson SA, Witt DM. Accuracy of ICD-10 codes for identifying hospitalizations for acute anticoagulation therapy-related bleeding events. *Thromb Res*. 2019;181(July):71-76. doi:10.1016/j.thromres.2019.07.021
12. Gergi M, Wilkinson K, Koh I, et al. The relative risk of bleeding after medical hospitalization: the medical inpatient thrombosis and hemorrhage study. *J Thromb Haemost*. 2023;21(3):513-521. doi:10.1016/j.jtha.2022.11.023
13. Kaatz S, Ahmad D, Spyropoulos AC, Schulman S, Subcommittee on Control of Anticoagulation. Definition of clinically relevant non-major bleeding in studies of anticoagulants in atrial fibrillation and venous thromboembolic disease in non-surgical patients: communication from the SSC of the ISTH. *J Thromb Haemost*. 2015;13(11):2119-2126. doi:10.1111/jth.13140
14. Schulman S, Kearon C, Subcommittee on Control of Anticoagulation of the Scientific and Standardization Committee of the International Society on Thrombosis and Haemostasis. Definition of major bleeding in clinical investigations of antihemostatic medicinal products in non-surgical patients. *J Thromb Haemost*. 2005;3(4):692-694. doi:10.1111/j.1538-7836.2005.01204.x
15. National Cancer Institute (NCI). NCI Comorbidity Index 2021 Version. Division of Cancer Control & Population Sciences. 2021. Accessed June 1, 2022. https://healthcaredelivery.cancer.gov/seermedicare/considerations/macro-2021.html
16. Heagerty PJ, Lumley T, Pepe MS. Time-dependent ROC curves for censored survival data and a diagnostic marker. *Biometrics*. 2000;56(2):337-344. doi:10.1111/j.0006-341x.2000.00337.x
17. Carrier M, Abou-Nassar K, Mallick R, et al. Apixaban to Prevent Venous Thromboembolism in Patients with Cancer. *N Engl J Med*. 2019;380(8):711-719. doi:10.1056/NEJMoa1814468
18. Khorana AA, Soff GA, Kakkar AK, et al. Rivaroxaban for Thromboprophylaxis in High-Risk Ambulatory Patients with Cancer. *N Engl J Med*. 2019;380(8):720-728. doi:10.1056/NEJMoa1814630

© 2025 Li A et al. *JAMA Network Open*.

eTable 1. Organization Filters

**Define high-quality sites:**
Country = United States of America
“Green lit” by Cosmos for data completeness
**Define sites with at least 2 years of valid data and codes:**
Started data contribution before 1/1/2023
Have ≥2 years of data with ≥1,000 monthly billing final diagnosis codes
**Define site with adequate inpatient & outpatient encounters in oncology departments:**
Have >1,000 inpatient encounters AND >1,000 emergency department visit encounters AND >1,000 outpatient face-to-face encounters AND >10,000 encounters at hematology or oncology departments
**Define non-pediatric sites:**
Average age of patients in the organization must be >22 years

© 2025 Li A et al. *JAMA Network Open*.

eTable 2. Cohort Filters

**Define *International Classification of Diseases, Tenth Edition, Clinical Modification* (ICD-10-CM) codes for cancer**
C__.%, C01, C07, C12, C19, C20, C23, C33, C37, C52, C55, C58, C61, C73<br>D45, D45.%, D46.%, D47.0%, D47.1%, D47.3%, D47.4%<br>Exclude: C44.%, C4A.%, C7B.1% (non-melanomatous skin cancer)<br>Between 1/1/2017 and 1/1/2025
**Define eligible encounter type (Face-to-Face or F2F)**
For billing final diagnosis:<br>Encounter type = ‘Hospital Encounter’ AND (IsHospitalAdmission = 1 OR IsEdVisit = 1 OR IsHospitalOutpatientVisit = 1) OR Encounter type IN (‘Diagnostic Services’, ‘Infusion’, ‘Procedure Visit’)<br>For encounter diagnosis:<br>Encounter type IN (‘Office Visit’, ‘Telemedicine’, ‘Anticoagulation Visit’, ‘Consult’, ‘Evaluation’, ‘Follow-Up’, ‘Procedural Consult’, ‘Surgical Consult’, ‘Transplant Evaluation’, ‘Transplant Follow Up’, ‘Urgent Care’, ‘Walk-In’, ‘Tumor Board Conference’)
**Define eligible department type**
IsHospitalAdmission = 1 (inpatient) OR DepartmentSpecialty LIKE ‘%Hematology%’, ‘%Oncology%’, ‘%Radiation%’, ‘%Infusion%’, ‘%Intensive%’, ‘%Critical%’, ‘%Surgery%’, ‘%Ear, Nose, Throat%’, ‘%Otolaryngology%’, ‘%Gynecology%’, ‘%Urology%’ (medical or surgical departments that diagnose and treat cancer)<br>Exclude: ‘%Neurology%’, ‘%Pediatric%’, ‘%Neonatal%’, ‘%Prenatal%’, ‘%Laboratory%’
**Define cancer exclusion criteria**
Exclude patients with any cancer diagnosis between 1/1/2017 and 1/1/2018 or within 1 year from data contribution start date of organization<br>Keep patients that have >=2 cancer diagnosis codes (excluding secondary/metastatic and unspecified) more than 30 days apart between 1/1/2018 and 1/1/2024 and >= 1 year from data contribution start date of organization<br>Exclude patients with polycythemia or essential thrombocythemia diagnosis<br>Exclude patients whose first cancer is not their most common cancer within 1 year of diagnosis<br>Exclude patients whose age at cancer diagnosis is <18 or >100
**Define systemic therapy**
TherapeuticClass = ‘antineoplastics’ AND Mode = ‘inpatient’ or ‘outpatient’ (administered or prescribed)<br>Exclude: PharmaceuticalSubClass: ‘Dermatological%’ and ‘DMARD%’; SimpleGenericName: ‘BCG live%’, ‘sirolimus%’, ‘medroxyprogesterone%’, ‘megestrol%’, ‘iobenguane%’, ‘methoxsalen%’; Route: ‘intra-pyelocalyceal’, ‘intravesical’<br>Exclude: patients with inpatient, outpatient, or historical mode systemic therapies before cancer diagnosis date
**Define final cohort exclusion**

© 2025 Li A et al. *JAMA Network Open*.

Exclude patients if not having at least 1 inpatient or outpatient “face-to-face” encounter in the 12 months window before their systemic therapy start date
Exclude patients if not having at least 2 inpatient or outpatient “face-to-face” continuous follow-up (<6 months apart) encounters after their systemic therapy start date
Exclude patients if having a recorded death date before their systemic therapy start date
Exclude patients if having a known stage 0 cancer in the past 1 year before their systemic therapy start date
Exclude patients if having been prescribed with anticoagulant in the past 1 year before their systemic therapy start date
Exclude patients if having been diagnosed with acute VTE in the past 1 year before their systemic therapy start date
Exclude patients if missing body mass index (BMI) in the past 3 months before their systemic therapy start date
Exclude patients if missing complete blood count (CBC) components in the past 3 months before their systemic therapy start date

© 2025 Li A et al. *JAMA Network Open*.

eTable 3. Systemic Therapy Classifications

**Cytotoxic Chemotherapy:**
altretamine, asparaginase, azacitidine, bendamustine, bleomycin, busulfan, cabazitaxel, calaspargase, capecitabine, carboplatin, carmustine, chlorambucil, cisplatin, cladribine, clofarabine, cyclophosphamide, cytarabine, dacarbazine, dactinomycin, daunorubicin, decitabine, docetaxel, doxorubicin, epirubicin, eribulin, etoposide, floxuridine, fludarabine, fluorouracil, gemcitabine, hydroxyurea, idarubicin, ifosfamide, irinotecan, ixabepilone, lomustine, lurbinectedin, mechlorethamine, melphalan, mercaptopurine, methotrexate, mitomycin, mitoxantrone, nelarabine, omacetaxine, oxaliplatin, paclitaxel, pegaspargase, pemetrexed, pentostatin, porfimer, pralatrexate, procarbazine, streptozocin, temozolomide, teniposide, thioguanine, thiotepa, topotecan, trabectedin, trifluridine, valrubicin, vinblastine, vincristine, vinorelbine
**Targeted Therapy**
abemaciclib, acalabrutinib, adagrasib, afatinib, aflibercept, alectinib, alemtuzumab, alpelisib, amivantamab, arsenic, asciminib, avapritinib, axitinib, belantamab, belinostat, belzutifan, bevacizumab, bexarotene, binimetinib, blinatumomab, bortezomib, bosutinib, brentuximab, brigatinib, cabozantinib, capivasertib, capmatinib, carfilzomib, ceritinib, cetuximab, cobimetinib, copanlisib, crizotinib, dabrafenib, dacomitinib, daratumumab, dasatinib, demcizumab, dostarlimab, duvelisib, elacestrant, elotuzumab, elranatamab, enasidenib, encorafenib, enfortumab, entrectinib, epcoritamab, erdafitinib, erlotinib, everolimus, fedratinib, fruquintinib, futibatinib, gefitinib, gemtuzumab, gilteritinib, glasdegib, ibrutinib, ibritumomab, idelalisib, imatinib, infigratinib, inotuzumab, isatuximab, ivosidenib, ixazomib, lapatinib, larotrectinib, lenalidomide, lenvatinib, loncastuximab, lorlatinib, margetuximab, midostaurin, mirvetuximab, mobocertinib, mogamulizumab, momelotinib, mosunetuzumab, necitumumab, neratinib, nilotinib, niraparib, nirogacestat, obinutuzumab, ofatumumab, olaparib, olaratumab, olutasidenib, osimertinib, pacritinib, palbociclib, pamrevlumab, panitumumab, panobinostat, pazopanib, pemigatinib, pertuzumab, pexidartinib, pirtobrutinib, polatuzumab, pomalidomide, ponatinib, pralsetinib, quizartinib, ramucirumab, regorafenib, ribociclib, ripretinib, rituximab, romidepsin, rucaparib, ruxolitinib, sacituzumab, selinexor, selpercatinib, selumetinib, siltuximab, sonidegib, sorafenib, sotorasib, sunitinib, tafasitamab, tagraxofusp, talazoparib, talquetamab, tazemetostat, tebentafusp, teclistamab, telotristat, temsirolimus, tepotinib, thalidomide, tisotumab, tivozanib, tositumomab, trametinib, trastuzumab, tremelimumab, tretinoin, tucatinib, umbralisib, upifitamab, vandetanib, veliparib, vemurafenib, venetoclax, vismodegib, vorinostat, zanubrutinib, glofitamab, lazertinib, repotrectinib, tarlatamab, vorasidenib
**Endocrine Therapy**
abarelix, abiraterone, anastrozole, apalutamide, bicalutamide, cyproterone, darolutamide, degarelix, enzalutamide, estramustine, exemestane, flutamide, fulvestrant, goserelin, histrelin, letrozole, leuprolide, mitotane, nilutamide, relugolix, tamoxifen, testolactone, toremifene, triptorelin
**Immune Checkpoint Inhibitor**
atezolizumab, avelumab, cemiplimab, durvalumab, ipilimumab, nivolumab, pembrolizumab, toripalimab
**Immune-CAR-T or Other: Excluded from current study**
axicabtagene, brexucabtagene, ciltacabtagene, idecabtagene, lisocabtagene, nadofaragene, talimogene, tisagenlecleucel<br>aldesleukin, interferon, peginterferon, ropeginterferon, sipuleucel, lifileucel

© 2025 Li A et al. *JAMA Network Open*.

eTable 4. Outcome Filters

**Define ICD-10-CM codes for VTE and bleeding:**
**VTE:**<br>**Acute PE:** I26.02, I26.09, I26.92, I26.93, I26.94, I26.99<br>**Acute LE-DVT:** I80.10, I80.11, I80.12, I80.13, I80.201, I80.202, I80.203, I80.209, I80.211, I80.212, I80.213, I80.219, I80.221, I80.222, I80.223, I80.229, I80.231, I80.232, I80.233, I80.239, I80.241, I80.242, I80.243, I80.249, I80.251, I80.252, I80.253, I80.259, I80.291, I80.292, I80.293, I80.299, I82.220, I82.401, I82.402, I82.403, I82.409, I82.411, I82.412, I82.413, I82.419, I82.421, I82.422, I82.423, I82.429, I82.431, I82.432, I82.433, I82.439, I82.441, I82.442, I82.443, I82.449, I82.451, I82.452, I82.453, I82.459, I82.461, I82.462, I82.463, I82.469, I82.491, I82.492, I82.493, I82.499, I82.4Y1, I82.4Y2, I82.4Y3, I82.4Y9, I82.4Z1, I82.4Z2, I82.4Z3, I82.4Z9<br>**Acute UE-DVT:** I82.210, I82.290, I82.621, I82.622, I82.623, I82.629, I82.A11, I82.A12, I82.A13, I82.A19, I82.B11, I82.B12, I82.B13, I82.B19, I82.C11, I82.C12, I82.C13, I82.C19, I82.601, I82.602, I82.603, I82.609<br>**SPVT:** I82.0, I82.1, I82.3, I82.890, I82.90, I80.3, I80.8, I80.9, I63.6, I67.6 (not used in current study)
**Bleeding:**<br>**Intracranial hemorrhage:** I60.00, I60.01, I60.02, I60.10, I60.11, I60.12, I60.2, I60.20, I60.21, I60.22, I60.30, I60.31, I60.32, I60.4, I60.50, I60.51, I60.52, I60.6, I60.7, I60.8, I60.9, I61.0, I61.1, I61.2, I61.3, I61.4, I61.5, I61.6, I61.8, I61.9, I62.00, I62.01, I62.02, I62.03, I62.1, I62.9, S06.340A, S06.341A, S06.342A, S06.343A, S06.344A, S06.345A, S06.346A, S06.347A, S06.348A, S06.349A, S06.350A, S06.351A, S06.352A, S06.353A, S06.354A, S06.355A, S06.356A, S06.357A, S06.358A, S06.359A, S06.360A, S06.361A, S06.362A, S06.363A, S06.364A, S06.365A, S06.366A, S06.367A, S06.368A, S06.369A, S06.370A, S06.371A, S06.372A, S06.373A, S06.374A, S06.375A, S06.376A, S06.377A, S06.378A, S06.379A, S06.380A, S06.381A, S06.382A, S06.383A, S06.384A, S06.385A, S06.386A, S06.387A, S06.388A, S06.389A, S06.4X0A, S06.4X1A, S06.4X2A, S06.4X3A, S06.4X4A, S06.4X5A, S06.4X6A, S06.4X7A, S06.4X8A, S06.4X9A, S06.4XAA, S06.5X0A, S06.5X1A, S06.5X2A, S06.5X3A, S06.5X4A, S06.5X5A, S06.5X6A, S06.5X7A, S06.5X8A, S06.5X9A, S06.6X0A, S06.6X1A, S06.6X2A, S06.6X3A, S06.6X4A, S06.6X5A, S06.6X6A, S06.6X7A, S06.6X8A, S06.6X9A<br>**Intraarticular:** M25.00, M25.011, M25.012, M25.019, M25.021, M25.022, M25.029, M25.031, M25.032, M25.039, M25.041, M25.042, M25.049, M25.051, M25.052, M25.059, M25.061, M25.062, M25.069, M25.071, M25.072, M25.073, M25.074, M25.075, M25.076, M25.08<br>**Intraocular:** H05.231, H05.232, H05.233, H05.239, H21.0, H31.301, H31.302, H31.303, H31.309, H31.311, H31.312, H31.313, H31.319, H31.411, H31.412, H31.413, H31.419, H35.60, H35.61, H35.62, H35.63, H35.731, H35.732, H35.733, H35.739, H43.10, H43.11, H43.12, H43.13, H44.81, H47.021, H47.022, H47.023, H47.029<br>**Lower GI:** K50.011, K50.111, K50.811, K50.911, K51.011, K51.211, K51.311, K51.411, K51.511, K51.811, K51.911, K55.21, K57.01, K57.11, K57.13, K57.21, K57.31, K57.33, K57.41, K57.51, K57.53, K57.81, K57.91, K57.93, K62.5, K63.81, K92.1, K92.2, K94.01, K94.11<br>**Upper GI:** I85.01, I85.11, K22.11, K22.6, K25.0, K25.2, K25.4, K25.6, K26.0, K26.2, K26.4, K26.6, K27.0, K27.2, K27.4, K27.6, K28.0, K28.2, K28.4, K28.6, K29.01, K29.21, K29.31, K29.41, K29.51, K29.61, K29.71, K29.81, K29.91, K31.811, K31.82, K92.0, K94.21, K94.31<br>**Pericardial:** I23.0, I31.2, S26.00XA, S26.01XA, S26.020A, S26.021A, S26.022A, S26.09XA

© 2025 Li A et al. *JAMA Network Open*.

**Pulmonary:** J95.01, R04.1, R04.2, R04.89, R04.9<br>**Retroperitoneal:** K66.1<br>**Soft Tissue:** M79.81<br>**Thoracic:** J94.2, S27.1XXA<br>**Ear:** H61.121, H61.122, H61.123, H61.129, H92.20, H92.21, H92.22, H92.23<br>**Genitourinary:** N30.41, N42.1, N99.510, N99.520, N99.530, R31.0<br>**Gynecologic:** O71.7, N83.6, N83.7, N93.8, N93.9<br>**Miscellaneous:** R58
**Define eligible encounter type:**
For billing final diagnosis:<br>Encounter type = ‘Hospital Encounter’ AND (IsHospitalAdmission = 1 OR IsEdVisit = 1 OR IsHospitalOutpatientVisit = 1)<br>OR Encounter type IN (‘Diagnostic Services’, ‘Infusion’, ‘Procedure Visit’)<br>For encounter diagnosis:<br>Encounter type IN (‘Office Visit’, ‘Telemedicine’, ‘Anticoagulation Visit’, ‘Consult’, ‘Evaluation’, ‘Follow-Up’, ‘Procedural Consult’, ‘Surgical Consult’, ‘Transplant Evaluation’, ‘Transplant Follow Up’, ‘Urgent Care’, ‘Walk-In’, ‘Tumor Board Conference’)
**Define outcome criteria:**
**VTE:** first Acute PE, Acute LE-DVT, Acute UE-DVT after index date from<br>Any inpatient face-to-face encounter<br>2+ outpatient face-to-face encounters that are >30 and <365 days apart<br>**Bleeding:** first bleeding event after index date from<br>Any inpatient face-to-face encounter

Abbreviations: ICD-10-CM, *International Classification of Diseases, Tenth Edition, Clinical Modification*; VTE, venous thromboembolism; PE, pulmonary embolism; LE-DVT, lower extremity deep vein thrombosis; UE-DVT, upper extremity deep vein thrombosis; SPVT, splanchnic vein thromboses; GI, gastrointestinal

© 2025 Li A et al. *JAMA Network Open*.

eTable 5. Baseline Variables

Variable	Criteria	Lookback Window
Age		At diagnosis
Sex		At diagnosis
Race/Ethnicity		At diagnosis
Marital status		At diagnosis
State		At diagnosis
Blood type		At diagnosis
RUCA code		At diagnosis
SVI ranking		At diagnosis
Cancer type	Condensed into 26 categories	At diagnosis
Cancer stage	Highest value	12 months before treatment
Treatment regimen	See Supplemental Table 3	Within 30 d of 1st treatment
Anticoagulant	TherapeuticClass = 'Anticoagulants'<br>Exclude: 'Anticoagulants – Citrate-based, Heparins'	12 months before treatment
Antiplatelet	TherapeuticClass = 'Antiplatelet Drugs'<br>Exclude: SimpleGenericName LIKE '%Aspirin%'	12 months before treatment
CYP3A4	GenericName: %adagrasib%, %apalutamide%, %carbamazepine%, %cobicistat%, %dronedarone%, %encorafenib%, %enzalutamide%, %erythromycin%, %fosphenytoin%, %isavuconazonium%, %itraconazole%, %john%, %ketoconazole%, % levoketoconazole%, %mifepristone%, %mitotane%, %nirmatrelvir%, %phenobarbital%, %phenytoin%, %posaconazole%, %primidone%, %rifamycin%, %rifampin%, %ritonavir%, %tucatinib%, %verapamil%, %wort%<br>Route: Oral, injection, intravenous	12 months before treatment
Weight, height, body mass index	No date limit for Height	-90 d to +7 d of treatment
Recent prolonged hospitalization	Admission >3 days ending before treatment<br>Admission >3 days ending after treatment	3 months before treatment
History of VTE	See Supplemental Table 4 for Acute VTE<br><br>Historic VTE: Z86.711, Z86.718<br>Chronic PE: I27.82<br>Chronic DVT: I82.211, I82.221, I82.291, I82.501, I82.502, I82.503, I82.509, I82.511, I82.512, I82.513, I82.519, I82.521, I82.522, I82.523, I82.529, I82.531, I82.532, I82.533,	**Acute:** PE, LE-DVT, UE-DVT, or SPVT within 12 months pre-treatment<br>**Remote:** Acute VTE >12 months pre-treatment, or any Chronic PE/DVT or

© 2025 Li A et al. *JAMA Network Open*.

	I82.539, I82.541, I82.542, I82.543, I82.549, I82.551, I82.552, I82.553, I82.559, I82.561, I82.562, I82.563, I82.569, I82.591, I82.592, I82.593, I82.599, I82.5Y1, I82.5Y2, I82.5Y3, I82.5Y9, I82.5Z1, I82.5Z2, I82.5Z3, I82.5Z9, I82.701, I82.702, I82.703, I82.709, I82.721, I82.722, I82.723, I82.729, I82.891, I82.91, I82.A21, I82.A22, I82.A23, I82.A29, I82.B21, I82.B22, I82.B23, I82.B29, I82.C21, I82.C22, I82.C23, I82.C29	Historic VTE before treatment
History of bleeding	See Supplemental Table 4 for Bleeding	**Acute:** Bleeding within 12 months pre-treatment<br>**Remote:** Bleeding >12 months pre-treatment
CBC:<br>1. WBC<br>2. Hgb<br>3. Plt	LOINC:<br>1. 804-5, 6690-2, 12227-5, 26464-8, 33256-9, 46498-9<br>2. 718-7, 14775-1, 20509-6, 30313-1, 30350-3, 30351-1, 30352-9, 55782-7, 76768-1, 76769-9, 97556-5, 97550-8, 97555-7<br>3. 777-3, 778-1, 13056-7, 26515-7, 26516-5, 49497-1, 74775-8, 74464-9, 97995-5	-90 d to +7 d of treatment
CMP:<br>1. Cr<br>2. Alb<br>3. ALT<br>4. TB	LOINC:<br>1. 2160-0, 21232-4, 38483-4<br>2. 1751-7, 2862-1, 61151-7, 61152-5, 76631-1, 77148-5, 101198-0, 103577-3<br>3. 1742-6, 1743-4, 1744-2, 76625-3, 77144-4<br>4. 1975-2, 42719-5, 59827-6, 59828-4	-90 d to +7 d of treatment
Metastatic Disease	ICD: C78.%, C79.%, C80.%, C7B.%	12 months before treatment
Metastatic Brain	ICD: C79.31	12 months before treatment
NCI Comorbidity Index (NCI-CI)	AcuteMi: I2[12]%<br>HistoryMi: I25.2<br>CHF: I09.9, I11.0, I13.0, I13.2, I25.5, I42.0, I43, P29.0, I42.[5-9], I50%<br>PVD: I7[0-1]%, I73.[189]%, 77.1, I79.[02], K55.[189], Z95.[8-9]%<br>CVD: G4[56]%, H34.0%, I6%<br>COPD: I27.[89]%, J[46][0-7]%, J68.4, J70.[13]<br>Dementia: F0[0-3]%, F05.1, G30%, G31.1<br>Paralysis: G04.1, G11.4, G80.[12], G8[12]%, G83.[0-49]%<br>Diabetes: E1[013].[01689]%<br>DiabetesComp: E1[013].[2-57]%<br>RenalDisease: I12.0, I13.1%, N0[35].[2-7], N1[8-9]%, N25.0, Z49.[0-2], Z94.0, Z99.2<br>MildLiverDisease: B18%, K70.[0-39]%, K71.[3-57]%, K7[34]%, K76.[02-4]%, Z94.4<br>LiverDisease: I85.[09]%, I86.4, I98.2, K70.4%, K71.1%, K72.[19]%, K76.[5-7]<br>Ulcers: K2[5-8]%<br>RheumDisease: M0[56]%, M31.5, M3[2-4]%, M35.[13]%, M36.0	12 months before treatment

© 2025 Li A et al. *JAMA Network Open*.

	HIVAIDS: B2[0-24]<br><br>NCI_CI = AcuteMi * 0.12624 + HistoryMi * 0.07999 + CHF * 0.64441 + PVD * 0.26232 + CVD * 0.27868 + COPD * 0.52487 + Dementia * 0.72219 + Paralysis * 0.39882 + GREATEST(Diabetes, DiabetesComp) * 0.29408 + RenalDisease * 0.47010 + GREATEST(MildLiverDisease, LiverDisease) * 0.73803 + Ulcers * 0.07506 + RheumDisease * 0.21905 + HIVAIDS * 0.58362	

Abbreviations: RUCA, Rural-Urban Commuting Area; SVI, Social Vulnerability Index; d, days; VTE, venous thromboembolism; PE, pulmonary embolism; DVT, deep vein thrombosis; LE-DVT, lower extremity deep vein thrombosis; UE-DVT, upper extremity deep vein thrombosis; SPVT, splanchnic vein thromboses; CBC, complete blood count; WBC, white blood cell; Hgb, hemoglobin; Plt, platelets; LOINC, Logical Observation Identifiers, Names, and Codes; CMP, complete metabolic panel; Cr, creatinine; Alb, albumin; ALT, alanine transaminase; TB, total bilirubin

© 2025 Li A et al. *JAMA Network Open*.

eTable 6. Detailed Predictor Definitions for EHR-CAT and Khorana Score

**Score Name**	Khorana Score	EHR-CAT
**Study Reference**	Khorana, *Blood* 2008	Li, *JCO* 2023
**Study Design**	Prospective	Retrospective
**Number of Patients in Derivation Cohort**	2,701	9,769
**VTE Outcome in Derivation Cohort**	60 at 3 mo (2.2%)	590 at 6 mo (6.2%)
**External Validation (c Statistic)**	0.57-0.64	0.68-0.71
**Risk Scores (predicted VTE at 6 months)**	0: 3%<br>1: 6%<br>2: 8%<br>≥3: 11%	≤0: 1%<br>1: 3%<br>2: 5%<br>3: 7%<br>4: 9%<br>≥5: 10-13%
**Risk Groups (predicted VTE at 6 months)**	<2: 4%<br>≥2: 9%	<3: 3%<br>≥3: 10%
**Risk Score Assignment**		
**Cancer Type**		
- Lung, kidney, bladder, testicular, ovarian, uterine, cervical, lymphoma	+1	
- Pancreas, stomach	+2	
- Colorectal		+1
- Lung, kidney, bladder, testicular, ovarian, uterine<br>- Sarcoma, brain, myeloma, acute lymphoblastic leukemia<br>- Selective lymphoma: diffuse large B-cell lymphoma, primary central nervous system lymphoma, Burkitt lymphoma, precursor non-Hodgkin lymphoma, systemic T/natural killer-cell lymphoma		+2
- Pancreas, stomach<br>- Esophageal, biliary, gallbladder		+3
**Pre-chemotherapy leukocyte** $>11 \times 10^9$/L	+1	+1
**Pre-chemotherapy hemoglobin** <10 g/dL	+1	+1
**Pre-chemotherapy platelet** $\geq 350 \times 10^9$/L	+1	+1
**BMI** ≥35 kg/m^2	+1	+1
**Advanced cancer stage** (AJCC III-IV, or unstageable aggressive hematologic malignancy)		+1

© 2025 Li A et al. *JAMA Network Open*.

**History of VTE** lifetime		+1
**History of paralysis/immobility** in last 12 months		+1
**Recent hospitalization** >3 days in last 3 months		+1
**Targeted or endocrine therapy** (without cytotoxic chemotherapy or immune checkpoint inhibitor)		-1
**Asian or Pacific Islander**		-1

Abbreviations: VTE, venous thromboembolism; mo, month; AJCC, American Joint Committee on Cancer

© 2025 Li A et al. *JAMA Network Open*.

eTable 7. Risk of Bleeding Based on Clinical Trial Exclusion Criteria

	Cancer Patients	Bleeding at 6 months	
	N (column %)	N (row %)	HR (95% CI)
**Cohort Incidence**			
All Patients	732,594 (100%)	26,993 (3.7%)	
**Overall Risk Factors**			
None of Below	542,181 (74.0%)	13,196 (2.4%)	1
Any of Below	190,413 (26.0%)	13,797 (7.2%)	2.55 (2.51, 2.59)
**Individual Risk Factors**			
Acute Leukemia	13,977 (1.9%)	1,844 (13.2%)	2.33 (2.22, 2.44)
Primary Brain	16,110 (2.2%)	694 (4.3%)	1.50 (1.42, 1.59)
Metastatic Brain	20,974 (2.9%)	1,549 (7.4%)	2.17 (2.09, 2.26)
Bleed History <1 Year	96,907 (13.2%)	8,083 (8.3%)	2.24 (2.20, 2.29)
Platelet $<50\times10^9$/L	14,349 (2.0%)	1,824 (12.7%)	1.87 (1.78, 1.95)
Alanine Transaminase >260 units/L	2,074 (0.3%)	156 (7.5%)	1.32 (1.17, 1.49)
Total Bilirubin >2.4 mg/dL	7,176 (1.0%)	727 (10.1%)	2.78 (2.62, 2.95)
eGFR <30 mL/min/1.73 m^2	17,708 (2.4%)	1,426 (8.1%)	2.19 (2.11, 2.29)
Weight <40 kg	2,412 (0.3%)	116 (4.8%)	1.43 (1.25, 1.62)
Anticoagulant (Stopped)	3,350 (0.5%)	213 (6.4%)	1.42 (1.28, 1.56)
Antiplatelet (Non-Aspirin)	17,419 (2.4%)	1,027 (5.9%)	1.67 (1.59, 1.74)
Strong CYP3A4 Inducer/Inhibitor	12,667 (1.7%)	660 (5.2%)	1.27 (1.21, 1.34)

Abbreviations: HR, hazard ratio; CI, confidence interval; eGFR, estimated glomerular filtration rate

© 2025 Li A et al. *JAMA Network Open*.

eTable 8. Performance of EHR-CAT vs Khorana Score for VTE at 6 Months After Exclusion for Bleeding Risk

	Cancer Patients	VTE at 6 months			PE/LE-DVT at 6 months		
	N (column %)	N (row %)	HR (95% CI)	TD-ROC	N (row %)	HR (95% CI)	TD-ROC
**Cohort Incidence**							
	542,181 (100.0%)	22,592 (4.2%)			18,328 (3.4%)		
**EHR-CAT Score**							
0-	200,759 (37.0%)	2,575 (1.3%)	1	**0.705** (0.704-0.707)	1,904 (0.9%)	1	**0.715** (0.712-0.718)
1	92,429 (17.0%)	3,092 (3.3%)	2.14 (2.06-2.21)		2,397 (2.6%)	2.11 (2.03-2.19)	
2	80,378 (14.8%)	3,722 (4.6%)	3.06 (2.96-3.16)		2,956 (3.7%)	3.11 (3.00-3.23)	
3	74,010 (13.7%)	4,467 (6.0%)	4.09 (3.96-4.22)		3,644 (4.9%)	4.22 (4.07-4.37)	
4	54,067 (10.0%)	4,318 (8.0%)	5.46 (5.28-5.64)		3,624 (6.7%)	5.70 (5.50-5.91)	
5+	40,538 (7.5%)	4,418 (10.9%)	7.67 (7.42-7.94)		3,803 (9.4%)	8.17 (7.87-8.47)	
**Khorana Score**							
0	222,677 (41.1%)	5,338 (2.4%)	1	**0.631** (0.628-0.634)	4,173 (1.9%)	1	**0.638** (0.632-0.638)
1	184,795 (34.1%)	7,734 (4.2%)	1.65 (1.61-1.69)		6,213 (3.4%)	1.67 (1.63-1.72)	
2	95,346 (17.6%)	5,941 (6.2%)	2.55 (2.48-2.62)		4,958 (5.2%)	2.64 (2.57-2.72)	
3+	39,363 (7.3%)	3,579 (9.1%)	3.62 (3.50-3.74)		2,984 (7.6%)	3.70 (3.57-3.83)	

Abbreviations: VTE, venous thromboembolism; PE, pulmonary embolism; LE-DVT, lower extremity deep vein thrombosis; HR, hazard ratio; CI, confidence interval; TD-ROC, time-dependent receiver operating characteristic

© 2025 Li A et al. *JAMA Network Open*.

eTable 9. Performance of EHR-CAT in Prespecified Subgroups

	Cancer Patients	VTE at 6 months		PE/LE-DVT at 6 months	
	N (column %)	N (row %)	TD-ROC	N (row %)	TD-ROC
**Age**					
18-55	179,619 (24.5%)	7,810 (4.3%)	0.700	5,657 (3.1%)	0.724
56-65	200,342 (27.3%)	9,779 (4.9%)	0.701	7,960 (4.0%)	0.715
66-75	219,488 (30.0%)	10,913 (5.0%)	0.695	9,344 (4.3%)	0.702
76+	133,145 (18.2%)	5,997 (4.5%)	0.689	5,380 (4.0%)	0.688
**Sex**					
Male	307,430 (42.0%)	16,249 (5.3%)	0.669	13,451 (4.4%)	0.675
Female	404,527 (58.0%)	18,249 (4.3%)	0.715	14,889 (3.5%)	0.730
**Race**					
White	583,047 (79.6%)	27,190 (4.7%)	0.698	22,543 (3.9%)	0.708
Black	94,269 (12.9%)	5,299 (5.6%)	0.688	4,262 (4.5%)	0.702
Asian	25,634 (3.5%)	706 (2.8%)	0.695	524 (2.0%)	0.711
Other	29,644 (4.0%)	1,304 (4.4%)	0.682	1,012 (3.4%)	0.695
**Ethnicity**					
Non-Hispanic	663,433 (90.6%)	31,484 (4.7%)	0.698	26,018 (3.9%)	0.708
Hispanic	48,266 (6.6%)	2,129 (4.4%)	0.695	1,634 (3.4%)	0.711

Abbreviations: VTE, venous thromboembolism; PE, pulmonary embolism; LE-DVT, lower extremity deep vein thrombosis; TD-ROC, time-dependent receiver operating characteristic

© 2025 Li A et al. *JAMA Network Open*.

eTable 10. Performance of EHR-CAT in 10 Randomly Selected Health Systems

	Cancer Patients	VTE at 6 months		PE/LE-DVT at 6 months		Top 3 Cancers
	N	N (row %)	TD-ROC	N (row %)	TD-ROC	
**Site**						
1	4,999	255 (5.1%)	0.697	229 (4.6%)	0.693	breast, lung, colorectal
2	6,607	294 (4.4%)	0.674	247 (3.7%)	0.671	breast, lung, prostate
3	9,386	324 (3.5%)	0.759	271 (2.9%)	0.762	breast, lung, colorectal
4	6,984	280 (4.0%)	0.698	226 (3.2%)	0.718	breast, lung, prostate
5	6,277	293 (4.7%)	0.643	240 (3.8%)	0.657	breast, lung, colorectal
6	6,711	318 (4.7%)	0.676	269 (4.0%)	0.682	breast, lung, colorectal
7	13,358	709 (5.3%)	0.714	590 (4.4%)	0.719	breast, lung, head/neck
8	1,630	79 (4.8%)	0.744	65 (4.0%)	0.750	breast, lung, colorectal
9	10,602	658 (6.2%)	0.682	534 (5.0%)	0.696	breast, lung, colorectal
10	22,152	1,022 (4.6%)	0.702	883 (4.0%)	0.707	breast, lung, colorectal
Overall	88,706	4,232 (4.8%) (3.5-6.2)	0.699 (0.643-0.759)	3,554 (4.0%) (2.9-5.0)	0.706 (0.657-0.762)	breast, lung, colorectal

Abbreviations: VTE, venous thromboembolism; PE, pulmonary embolism; LE-DVT, lower extremity deep vein thrombosis; TD-ROC, time-dependent receiver operating characteristic

© 2025 Li A et al. *JAMA Network Open*.

eTable 11. Exploratory Multivariable Cox Regression for EHR-CAT and Khorana Score Individual Predictors

	Cancer Patients		EHR-CAT		Khorana Score	
	P	N (column %)	VTE HR (95% CI)	PE/LE-DVT HR (95% CI)	VTE HR (95% CI)	PE/LE-DVT HR (95% CI)
**EHR-CAT Cancer Class**						
Low Risk	0	401,096 (54.8%)	1	1		
Intermediate Risk	1	59,852 (8.2%)	1.27 (1.24-1.31)	1.42 (1.38-1.47)		
High Risk	2	220,294 (30.1%)	1.71 (1.68-1.75)	1.91 (1.87-1.95)		
Very High Risk	3	51,352 (7.0%)	2.58 (2.51-2.66)	2.89 (2.89-3.07)		
**Khorana Score Cancer Class**						
Low Risk	0	493,268 (67.3%)			1	1
High Risk	1	203,993 (27.8%)			1.83 (1.80-1.86)	1.88 (1.84-1.91)
Very High Risk	2	35,333 (4.8%)			3.28 (3.19-3.37)	3.51 (3.40-3.62)
**Body Mass Index (BMI)**						
BMI ≥ 35	1	113,333 (15.5%)	1.33 (1.30-1.36)	1.37 (1.33-1.39)	1.27 (1.24-1.30)	1.30 (1.27-1.32)
**Complete Blood Count**						
WBC > 11 x 10^9/L	1	117,748 (16.1%)	1.28 (1.25-1.30)	1.29 (1.26-1.32)	1.34 (1.31-1.36)	1.33 (1.31-1.36)
Hgb < 10 g/dL	1	105,419 (14.4%)	1.16 (1.13-1.18)	1.11 (1.09-1.14)	1.44 (1.41-1.47)	1.37 (1.34-1.40)
Plt ≥ 350 x 10^9/L	1	117,572 (16.0%)	1.07 (1.05-1.10)	1.08 (1.05-1.10)	1.21 (1.19-1.24)	1.22 (1.20-1.25)
**Cancer Stage**						
Stage I	0	79,368 (10.8%)	1	1		
Stage II	0	44,438 (6.1%)	1.41 (1.34-1.49)	1.41 (1.33-1.50)		
Stage III	1	58,126 (7.9%)	1.82 (1.74-1.91)	1.82 (1.73-1.92)		
Stage IV	1	60,700 (8.3%)	2.85 (2.73-2.99)	2.96 (2.81-3.11)		
Metastatic ICD code	1	145,634 (19.9%)	2.71 (2.60-2.83)	2.80 (2.67-2.94)		
Unstageable	0	119,956 (16.4%)	1.61 (1.54-1.68)	1.48 (1.41-1.56)		
Missing/Unknown	0	224,372 (30.6%)	1.69 (1.62-1.76)	1.71 (1.63-1.79)		

© 2025 Li A et al. *JAMA Network Open*.

Therapy Type						
Cytotoxic Chemo	0	446,048 (60.9%)	1	1		
Immune Checkpoint Inhibitor	0	47,915 (6.5%)	0.82 (0.80-0.85)	0.89 (0.85-0.91)		
Targeted Therapy	-1	81,987 (11.2%)	0.80 (0.77-0.82)	0.89 (0.85-0.90)		
Endocrine Therapy	-1	156,644 (21.4%)	0.44 (0.43-0.46)	0.53 (0.50-0.54)		
**Race**						
White	0	583,047 (79.6%)	1	1		
Black	0	94,269 (12.9%)	1.30 (1.27-1.33)	1.25 (1.23-1.28)		
Asian or Pacific Islander	-1	25,634 (3.5%)	0.65 (0.62-0.69)	0.60 (0.57-0.64)		
Other/ Unknown	0	29,644 (4.0%)	0.92 (0.88-0.95)	0.87 (0.83-0.91)		
**Other Predictors**						
Hospitalization Last 3 Months	1	171,145 (23.4%)	1.28 (1.26-1.30)	1.28 (1.25-1.30)		
Paralysis Last Year	1	9,891 (1.4%)	1.47 (1.41-1.56)	1.64 (1.55-1.73)		
Chronic VTE Last Year or Acute >1 Year	1	23,474 (3.2%)	2.03 (1.99-2.09)	2.14 (2.06-2.21)		

Abbreviations: VTE, venous thromboembolism; PE, pulmonary embolism; LE-DVT, lower extremity deep vein thrombosis; HR, hazard ratio; CI, confidence interval; ICD, International Classification of Diseases

© 2025 Li A et al. *JAMA Network Open*.

eFigure 1. Incidence of VTE at 6 Months by Cancer Type

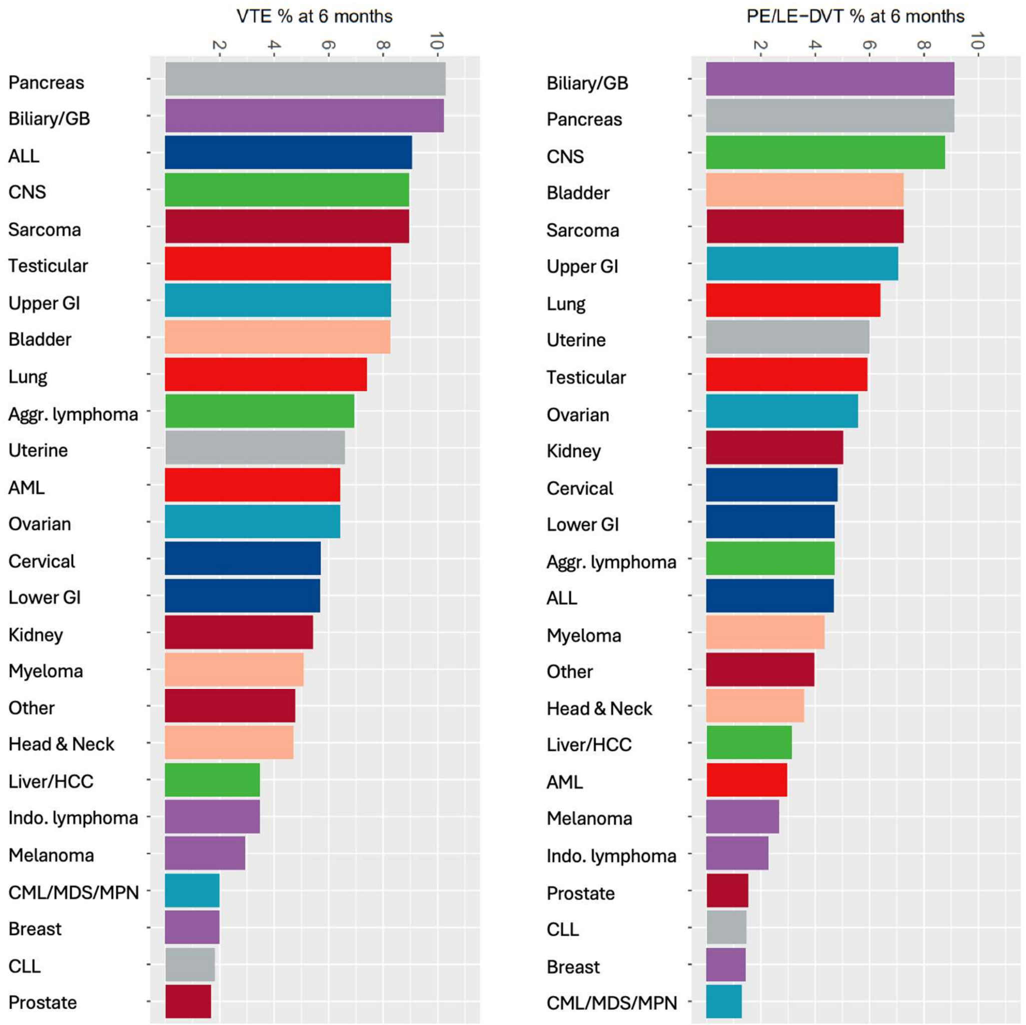

Abbreviations: VTE, venous thromboembolism; PE/LE-DVT, pulmonary embolism or lower extremity deep vein thrombosis; GB, gallbladder; ALL, acute lymphoblastic leukemia; CNS, central nervous system; GI, gastrointestinal; aggr., aggressive; AML, acute myeloid leukemia; HCC, hepatocellular carcinoma; indo., indolent; CML, chronic myeloid leukemia; MDS, myelodysplastic syndrome; MPN, myeloproliferative neoplasm; CLL, chronic lymphocytic leukemia

© 2025 Li A et al. *JAMA Network Open*.

eFigure 2. Calibration Plots of EHR-CAT vs Original Derivation Model

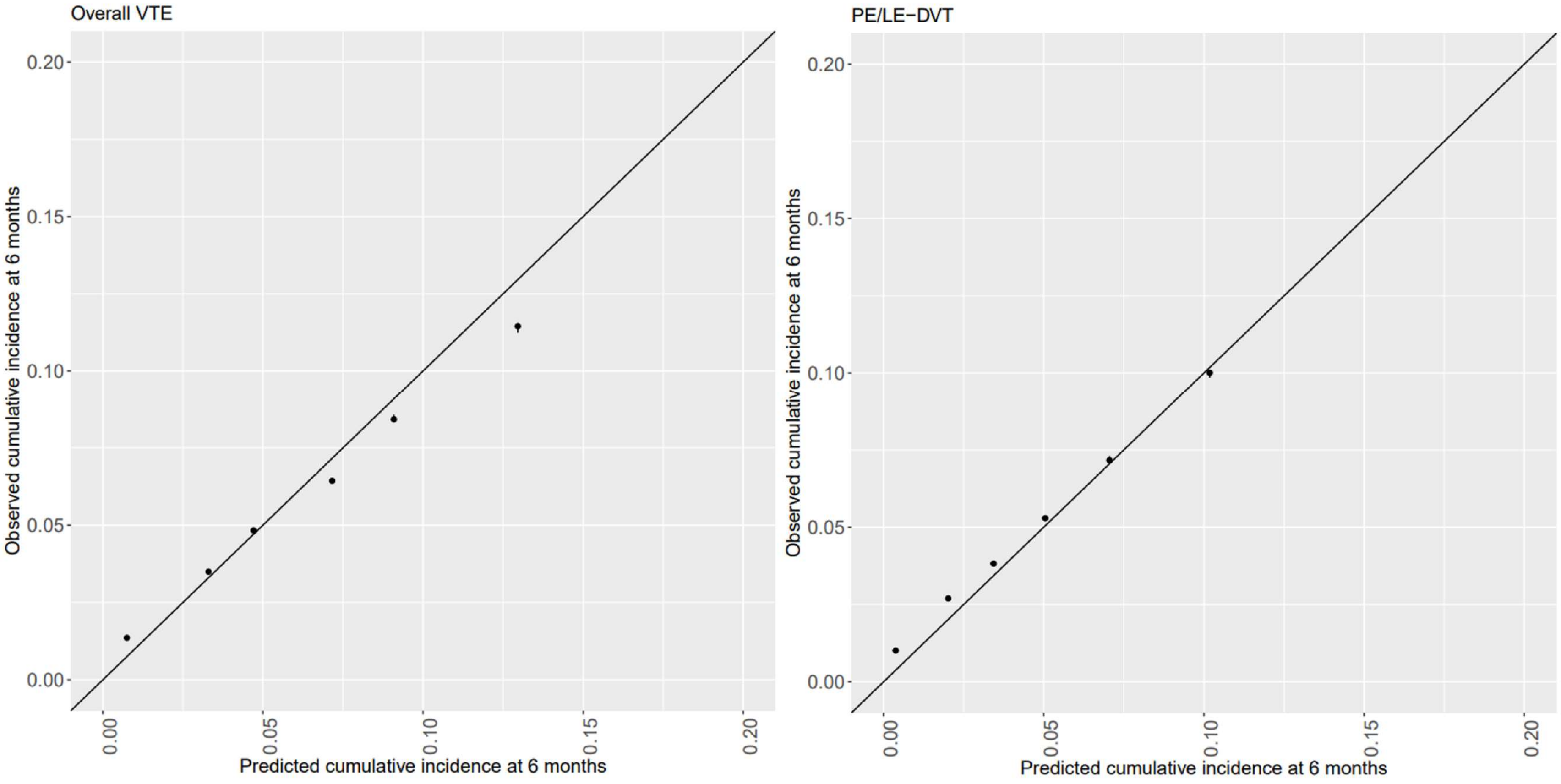

© 2025 Li A et al. *JAMA Network Open*.
