# Supplementary material for: Validation of a Risk Score for Cancer-Associated Thrombosis Using Nationwide EHR Data
Source: JAMA Netw Open. 2025 Nov 25;8(11):e2544428. doi: 10.1001/jamanetworkopen.2025.44428 (PMC12648341; doi:10.1001/jamanetworkopen.2025.44428)
Supplement: Supplement 2. — Data Sharing Statement [file jamanetwopen-e2544428-s002.pdf]

Data Sharing Statement

Li. Validation of a Risk Score for Cancer-Associated Thrombosis Using Nationwide EHR Data. *JAMA Netw Open*. Published November 25, 2025. doi:10.1001/jamanetworkopen.2025.44428

Data
Data available: Yes
Data types: Other (please specify)
Additional Information: De-identified database.
How to access data: The data belong to Epic Cosmos. https://cosmos.epic.com/request-access/
When available: With publication

Supporting Documents
Document types: None

Additional Information
Who can access the data: Researchers whose proposed use of the data has been approved by Epic.
Types of analyses: Detailed supplemental methods for cohort definition, data extraction, and analyses.
Mechanisms of data availability: Signed data access agreement between requester and Epic.
